# Harnessing M1-Polarized Macrophage-Derived Extracellular Vesicles and Artificial Nanovesicles for Targeted Cancer Drug Delivery

**DOI:** 10.3390/cells15110987

**Published:** 2026-05-27

**Authors:** Prakash Gangadaran, Sanjuda Subramaniyan, Ramya Lakshmi Rajendran, Chae Moon Hong, Kumari Swati, Saurabh Kumar Jha, Shazia Rashid, Byeong-Cheol Ahn

**Affiliations:** 1 Department of Nuclear Medicine, School of Medicine, Kyungpook National University, Daegu 41944, Republic of Korea; prakashg@knu.ac.kr (P.G.); ramyag@knu.ac.kr (R.L.R.); cmhong@knu.ac.kr (C.M.H.); 2 Cardiovascular Research Institute, Kyungpook National University, Daegu 41944, Republic of Korea; 3 Amity Institute of Biotechnology, Amity University Uttar Pradesh (AUUP), Noida 201313, India; sanjudasubramaniyan@gmail.com; 4 BK21 FOUR KNU Convergence Educational Program of Biomedical Sciences for Creative Future Talents, Department of Biomedical Sciences, School of Medicine, Kyungpook National University, Daegu 41944, Republic of Korea; 5 Department of Nuclear Medicine, Kyungpook National University Hospital, Daegu 41944, Republic of Korea; 6 Department of Biotechnology and Microbiology, SRM University, Sonipat 131023, India; sweetysoni.swati@gmail.com; 7 Department of Zoology, Kalindi College, University of Delhi, Delhi 110008, India; jhasaurabh017@gmail.com; 8 Department of Biotechnology Engineering and Food Technology, Chandigarh University, Mohali 140413, India

**Keywords:** macrophage-derived extracellular vesicles, tumor-associated macrophages (TAMs), artificial nanovesicles, tumor microenvironment, precision oncology

## Abstract

**Highlights:**

**What are the main findings?**

- M1-polarized macrophage-derived extracellular vesicles carry an innate dual therapeutic identity, simultaneously delivering cytotoxic and immunostimulatory cargo while homing to tumor tissue via surface-expressed integrins, CD44, and CD47, differentiating them mechanistically from conventional nanoparticle drug delivery systems.
- Artificial nanovesicles (ANVs) engineered from M1 macrophage membranes encapsulate key M1 surface properties and provide improved scalability and compositional control over natural EVs, though M1-specific evidence is still emerging, and current ANV principles are largely extrapolated from general nanomedicine frameworks.

**What are the implications of the main findings?**

- M1-derived EVs overcome the limitations of current traditional nanocarriers by actively homing to tumors, evading immunity, and reprogramming tumor-associated macrophages, shifting the cancer nanomedicine model from passive delivery to active, biologically driven therapy.
- Standardization of M1 polarization protocols, MISEV2023-compliant EV characterization, and systematic evaluation of biodistribution and off-target inflammatory safety are critical prerequisites for translating M1 macrophage-derived EV and ANV platforms from bench to clinical application.

**Abstract:**

Macrophage-derived extracellular vesicles (EVs) have emerged as promising biomimetic platforms for targeted cancer drug delivery due to their biocompatibility, immune-modulatory properties, and tumor-homing capabilities. Among macrophage subtypes, M1-polarized macrophages exhibit potent anti-tumor functions characterized by pro-inflammatory cytokine secretion, improved antigen presentation, and the ability to remodel the tumor microenvironment (TME). Utilizing these properties, M1-polarized macrophage-derived EVs serve as cell-free therapeutic systems capable of delivering bioactive cargo while simultaneously promoting anti-tumor immune responses. However, the clinical application of natural EVs is limited by low yield, heterogeneity, and challenges in large-scale production. Artificial nanovesicles (ANVs) have been developed to address these limitations, offering improved scalability, compositional control, and reproducibility. This review provides an overview of macrophage differentiation and polarization, with a focus on the immunological profile and anti-tumor mechanisms of M1-polarized macrophages. It further discusses current methodologies for EV isolation and ANV generation, along with cargo loading strategies that balance encapsulation efficiency and vesicle stability. In addition, this review also emphasizes their targeting approaches, cellular uptake pathways, and the intracellular trafficking mechanisms that influence delivery efficiency and therapeutic outcomes. Key challenges, including standardization, biological barriers, and functional consistency, are critically evaluated. Emerging strategies that integrate vesicle engineering with personalized medicine underscore the potential of these systems to advance precision oncology.

## 1. Introduction

Cancer remains one of the leading causes of morbidity and mortality worldwide, with therapeutic resistance, systemic toxicity, and poor target specificity continuing to limit the efficacy of current treatment modalities such as chemotherapy, radiotherapy, and even emerging targeted therapies [1]. A major contributor to such limitations is the tumor microenvironment (TME), a highly dynamic and heterogeneous ecosystem composed of stromal cells, immune cells, extracellular matrix components, and soluble signaling factors [2]. The TME actively regulates tumor progression, immune evasion, metastasis, and therapeutic response [3].

Among the diverse cellular components of the TME, the tumor-associated macrophages (TAMs) represent one of the most abundant and functionally versatile cell populations [4]. The high plasticity of macrophages enables them to adopt a spectrum of activation states in response to environmental cues [5]. Classically activated (M1-like) macrophages are characterized by pro-inflammatory, anti-tumorigenic functions, and alternatively activated (M2-like) macrophages typically promote tissue repair, immune suppression, angiogenesis, and tumor progression [6,7]. In many solid tumors, TAMs tend to shift to an M2-phenotype, contributing to immunosuppression, which facilitates tumor progression and therapeutic resistance [8]. Although macrophages are traditionally described based on M1/M2-phenotypes, it is increasingly recognized that macrophages are highly fluidic and can change their phenotypic states according to the local cytokine microenvironments [9]. Recent advances in immunoengineering and nanomedicine have highlighted how the function of macrophages can be reprogrammed and how their intrinsic biological properties can be utilized for better therapeutic outcomes [10]. In particular, M1-polarized macrophages are known to secrete pro-inflammatory cytokines, produce reactive oxygen species (ROS) and reactive nitrogen species (RNS), and enhance antigen presentation, thereby promoting anti-tumor immune responses [11,12]. However, challenges such as poor in vivo persistence, phenotypic instability, and difficulties in controlled delivery restrict the direct therapeutic application of macrophages [13].

An emerging cell-free therapeutic platform is the extracellular vesicles (EVs), which are lipid bilayer-enclosed nanostructures naturally secreted by cells that can transfer bioactive cargo, such as proteins, lipids, and nucleic acids, to recipient cells [14]. EVs derived from M1-polarized macrophages also have key immunomodulatory and anti-tumor properties of their parent cells, providing advantages like improved stability, reduced immunogenicity, and enhanced tissue penetration [15]. Their ability to interact with tumor cells and modulate the TME makes EVs good candidates for targeted drug delivery [16]. Despite their potential, the clinical translation of natural EVs is limited by large-scale production, heterogeneity, and cargo loading efficiency. These challenges can be addressed by developing artificial nanovesicles (ANVs), which mimic the structural and functional properties of natural EVs while offering greater control over composition, scalability, and therapeutic payload [8]. ANVs are engineered vesicle systems generated through physical or chemical methods, such as microfluidic fabrication or membrane extrusion, which can be further functionalized to enhance targeting specificity and delivery efficiency [17]. A promising strategy lies in integrating the immunological advantages of M1-polarized macrophage-derived vesicles with advanced nanotechnological approaches to create hybrid delivery systems capable of precise tumor targeting. In this way, such engineered systems not only enable efficient drug delivery but also actively modulate the TME toward an anti-tumor state, providing a synergistic dual therapeutic benefit [8].

This review provides a comprehensive overview of the emerging field of M1-polarized macrophage-derived EVs and ANVs in cancer therapy. Strategies for cargo loading and targeting delivery are examined, along with current challenges and future directions for clinical translation. Through a critical study of these emerging platforms, this review identifies the essential parameters required for the development of standardized, next-generation biomimetic vehicles for precision cancer therapy.

## 2. Types of Macrophages

### 2.1. Macrophage Differentiation and Plasticity

Macrophages are highly specialized innate immune cells that play vital roles in tissue homeostasis, host defense, and inflammation [18]. Their functional heterogeneity arises from both their developmental origin and their ability to dynamically respond to environmental cues [19]. Therefore, understanding the differentiation and plasticity of macrophages is crucial for utilizing them for their therapeutic potential, especially in cancer [20].

The primary sources from which macrophages originate are embryonic progenitors and bone marrow-derived monocytes [21]. Tissue-resident macrophages, such as microglia in the brain and Kupffer cells in the liver, originate during embryogenesis and maintain their populations during adulthood via self-renewal. In contrast, monocytes derived from hematopoietic stem cells in the bone marrow circulate in the blood and are recruited to peripheral tissues during inflammation or disease, where they differentiate into macrophages [22]. Monocyte-to-macrophage differentiation is regulated by two cytokines, namely macrophage colony-stimulating factor (M-CSF) and granulocyte-macrophage colony-stimulating factor (GM-CSF) [23]. M-CSF (or CSF-1) mainly drives homeostatic differentiation and survival, while GM-CSF is induced during inflammation to generate specialized macrophage populations and dendritic cells [24]. Such differentiation pathways are modulated by transcription factors such as PU1, IRF family members, and NF-κB, which coordinate lineage commitment and function [25]. One of the defining characteristics of macrophages is their plasticity, which allows them to reversibly transition between different activation states based on evolving local microenvironmental signals [25,26]. Unlike terminally differentiated cells with fixed, permanent roles, macrophages function along a continuous spectrum, allowing them to adapt to both physiological and pathological conditions. This plasticity is controlled by various signaling pathways, metabolic shifts, and epigenetic factors [27]. External stimuli activate intracellular signaling cascades, including the NF-κB, STAT, and MAPK pathways [28]. The functional shifting between M1- and M2-phenotypes is driven by metabolic reprogramming, where cellular energy production pathways are changed to meet specific functional needs [29]. M1-like macrophages, which defend against acute pathogens, undergo a transition to aerobic glycolysis even in the presence of oxygen (the Warburg effect), while M2-like macrophages, involved in tissue repair, rely on oxidative phosphorylation and fatty acid oxidation [8]. Epigenetic modifications, such as histone acetylation, methylation, and chromatin remodeling, are the key molecular mechanisms that allow macrophages to remember prior stimuli, a process termed “trained immunity.” This memory is packaged into the molecular cargo of EVs, which allows them to carry the polarized signature of the parent cell even after secretion [30]. In the TME, macrophage plasticity is particularly evident. Tumor-derived signals, including hypoxia, lactic acid accumulation, and immunosuppressive cytokines, can reprogram macrophages to phenotypes that support tumor growth, angiogenesis, and immune evasion [31].

While plasticity enables macrophages to be engineered for therapeutic applications, it also raises concerns regarding phenotypic instability, particularly in hostile microenvironments such as tumors [32]. To address such challenges, cell-free approaches, such as macrophage-derived EVs, are developed, which can retain functional characteristics of specific activation states without being affected by uncontrolled reprogramming that occurs in vivo. By using the immunological profile of an M1-polarized macrophage, these vesicles provide a predictable, stable alternative to cell-based therapies.

### 2.2. Phenotypic Spectrum: From Pro-Inflammatory M1 to Anti-Inflammatory M2

Macrophage activation has traditionally been classified into two polarized states: the classically activated M1-phenotype and the alternatively activated M2-phenotype. Although this framework provides the foundation for understanding the functional roles of macrophages, their activation is now widely known to exist on a dynamic spectrum, rather than in discrete states [33].

#### 2.2.1. M1-Polarized Macrophage Activation

M1-like macrophages are induced in response to pro-inflammatory stimuli, such as interferon-gamma (IFN-γ) and tumor necrosis factor-alpha (TNF-α). These signals activate key intracellular pathways, such as STAT1 and MAPK signaling cascades, leading to the expression of a transcription program associated with host defense and anti-tumor immunity [34]. M1-like macrophages are functionally characterized by the following: high production of pro-inflammatory cytokines, including interleukin-1β (IL-1β), IL-6, IL-12 and TNF-α, high expression of major histocompatibility complex class II (MHC-II) and co-stimulatory molecules (CD80, CD86), enhancing antigen presentation; induction of inducible nitric oxide synthase (iNOS), resulting in nitric oxide (NO) production from L-arginine, and generation of ROS via NADPH oxidase complexes [8].

#### 2.2.2. M2-Polarized Macrophage Activation

M2-like macrophages arise in response to anti-inflammatory and tissue-repair-associated signals, including in IL-4, IL-13, IL-10, and glucocorticoids [35]. These stimuli activate STAT6, PPAR-γ, and other transcriptional regulators, which promote an immunosuppressive, reparative phenotype [35,36]. M2 macrophages are functionally associated with the following: secretion of anti-inflammatory cytokines such as IL-10 and TGF-β, expression of scavenger receptors and markers such as CD163 and CD206, promotion of extracellular matrix remodeling and tissue repair, induction of angiogenesis through factors such as VEGF and matrix metalloproteinases (MMPs), and the upregulation of arginase-1 (Arg1), which competes with iNOS for L-arginine, effectively depleting the substrate needed for NO production and promoting polyamine synthesis for collagen deposition [8,37,38].

Although the M1/M2 classification is conceptually useful, the latest studies indicate that macrophage activation is far more complex. In vivo, macrophages exhibit hybrid or intermediate states shaped by a combination of signals with their microenvironment, rather than M1- or M2-phenotypes exclusively [39]. Underlying these phenotypes is the difference in metabolic reprogramming: M1-phenotype macrophages rely on aerobic glycolysis, supporting rapid energy production, and undergo a disrupted Krebs cycle, accumulating pro-inflammatory metabolites such as succinate and citrate, whereas M2-phenotype macrophages favor oxidative phosphorylation and fatty acid oxidation [40]. Metabolic byproducts of these states, such as lactate in the TME, further reinforce M2 polarization [41]. Although macrophage plasticity allows transition between phenotypes, it is often co-opted to favor tumor-promoting phenotypes in pathological conditions like cancer [42]. EVs derived from M1-polarized macrophages effectively bypass this metabolic subversion by the TME by delivering functional M1 cargo in a stable, cell-free format that can provide targeted anti-tumor responses [8].

## 3. M1-Polarized Macrophages Immunological Profile and Anti-Tumor Function

### 3.1. Surface Markers and Cytokine Profile of M1 Macrophages

M1-polarized macrophages are characterized by pro-inflammatory and immunostimulatory profiles. This phenotype is induced by stimuli like IFN-γ, lipopolysaccharide (LPS), and TNF-α, which collectively activate transcriptional programs mediated by NF-κB, STAT1, and interferon regulatory factors (IRFs) [43]. These signaling pathways coordinate the expression of distinct surface markers, cytokines, and effector molecules that promote the anti-tumor functions of M1 macrophages [44].

M1-like macrophages are known for their enhanced capacity for antigen presentation and T-cell activation. This is mediated through the upregulation of MHC-II molecules, which helps in the presentation of processed antigenic peptides to CD4+ T helper cells [45]. M1 activation also promotes MHC-I expression, enabling the priming of CD8+ cytotoxic T lymphocytes, which is particularly relevant in anti-tumor immunity [46]. M1-like macrophages also exhibit high expression of co-stimulatory molecules CD80 (B7-1) and CD86 (B7-2), which deliver secondary signals required for productive T cell priming and activation [47]. The surface phenotype of M1 macrophages is known to be their high expression of CD64/FcγRI (FcγRI), which enables antibody-dependent cellular cytotoxicity (ADCC), and CD197 (CCR7), a chemokine receptor that supports migration toward lymphoid tissues and coordination of adaptive immune responses [48]. TLR4 and TLR2 signaling, which are key upstream drivers of M1 polarization, sustain heightened sensitivity to pathogen-associated molecular patterns (PAMPs) and damage-associated molecular patterns (DAMPs) within the TME [8]. Collectively, this surface marker profile, which contains co-stimulatory ligands, Fc receptors, and chemokine receptors, places M1 macrophages as potent professional antigen-presenting cells (APCs) that can bridge innate and adaptive immunity [49].

M1-like macrophages are differentiated by their secretion of various pro-inflammatory cytokines that shape both local and systemic immune responses [50]. IL-12 is a key cytokine that drives Th1 polarization and IFN-γ production by T cells and natural killer (NK) cells [51]. TNF-α promotes inflammation, apoptosis of tumor cells, and activation of immune effector functions [52]. IL-6 and IL-1β are other cytokines that contribute to the amplification of inflammatory signaling and recruitment of immune cells [53]. Apart from cytokines, M1-like macrophages also produce chemokines such as CXCL9, CXCL10 and CXCL11, which are crucial for recruiting effector T cells into the TME [54]. M1-like macrophages also exert cytotoxic effects through the production of reactive and enzymatic mediators. One of the hallmarks of M1 activation is the upregulation of inducible iNOS, which catalyzes the production of NO [55]. M1-like macrophages also generate ROS via activation of the NADPH oxidase complex. Such reactive intermediates can directly induce DNA damage, mitochondrial dysfunction, and apoptosis in tumor cells and also enhance anti-tumor responses by modulating signaling pathways within the TME [56]. The molecular profile of M1-like macrophages is reflected in the composition of their secreted EVs. These M1-like macrophage-derived EVs act as carriers for pro-inflammatory cytokines, immunostimulatory proteins, and regulatory nucleic acids, including microRNAs (miRNAs), which can modulate gene expression in recipient cells [57]. Such vesicles can enhance antigen representation and immune activation, promote pro-inflammatory signaling in target cells, and reprogram tumor-associated macrophages toward an anti-tumor phenotype, which are key aspects of M1-like macrophage function.

### 3.2. Mechanisms of Anti-Tumor Activity in the Tumor Microenvironment

M1-like macrophages provide anti-tumor effects through a combination of direct cytotoxic mechanisms, immune activation, and reprogramming of the TME. Due to their diverse capabilities, M1-like macrophages act as primary drivers of anti-tumor immunity, simultaneously directing innate and adaptive defenses while suppressing signals that promote tumor growth [58].

**Figure 1:**
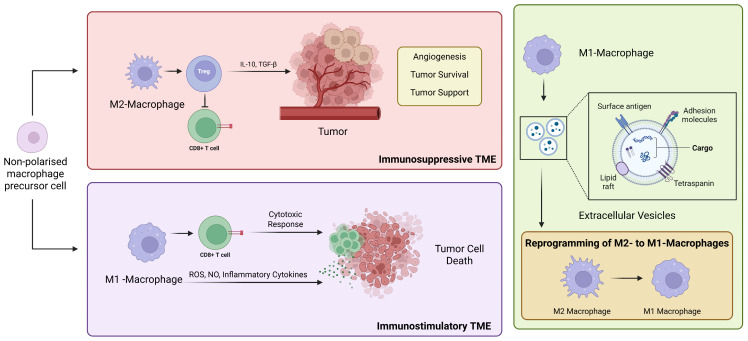
Reprogramming of the tumor microenvironment by M1-like macrophages and their extracellular vesicles. M2 macrophages promote an immunosuppressive tumor microenvironment (TME) via IL-10/TGF-β, supporting tumor growth, while M1-like macrophages induce an immunostimulatory TME through CD8+ T cell activation and inflammatory mediators, leading to tumor cell death. M1-like macrophage-derived extracellular vesicles (EVs) carry bioactive cargo and surface molecules that enable targeting and reprogramming of M2- to M1-phenotypes. TME: tumor microenvironment; EVs: extracellular vesicles; IL-10: interleukin-10; TGF-β: transforming growth factor-beta; Treg: regulatory T cell; ROS: reactive oxygen species; NO: nitric oxide. Created in BioRender. Subramaniyan, S. (2026) https://BioRender.com/pj1rbmc (accessed on 24 May 2026).

One of its primary mechanisms to eliminate tumor cells is by producing cytotoxic mediators, including ROS and RNS [59]. The upregulation of iNOS leads to the generation of NO, which causes DNA damage, disrupts mitochondrial function, and induces apoptosis in tumor cells [60]. ROS produced via NADPH oxidase activity causes oxidative stress, amplifying tumor cell death. In addition to reactive intermediates, M1-like macrophages secrete pro-inflammatory cytokines such as TNF-α, which can directly induce apoptotic and necroptotic pathways in the tumor [61]. Moreover, M1-like macrophages also function as professional APCs [62]. Increased expression of MHC-II and co-stimulatory molecules (CD80, CD86) allows tumor cells to effectively present antigens to CD4+ T cells, promoting their activation and differentiation into Th1 effector cells [63]. This process creates a positive feedback loop, where IFN-γ enhances M1-polarized macrophages, thereby amplifying anti-tumor immunity [64,65]. Beyond direct cytotoxicity, M1-like macrophages reprogram the TME to promote anti-tumor immunity. They counteract immunosuppressive TAMs by suppressing IL-10 and TGF-β, inhibiting regulatory T cell (Treg) function, and secreting CXCL9/CXCL10 to recruit effector immune cells [66]. Furthermore, M1-like macrophages can influence tumor vasculature and stromal remodeling by inhibiting angiogenic factors and modulating the extracellular matrix, contributing to reduced tumor growth. M1-like macrophages also exhibit anti-tumor activity through their interaction with other immune cell types [67]. Through cytokine and chemokine signaling, M1-like macrophages recruit and activate key immune cells, such as cytotoxic CD8+ T cells (for targeted killing), NK cells (for innate cytotoxicity), and dendritic cells (for immune priming) [68,69]. This coordinated immune network creates a highly inflammatory and cytotoxic microenvironment that is unfavorable for tumor survival [70]. M1-like macrophage-derived EVs suppress tumor progression by delivering bioactive cargo, such as cytokines, signaling proteins, and miRNAs, which induces apoptosis, enhances antigen presentation, and reprograms immunosuppressive macrophages within the TME [8]. Thus, M1-like macrophage-derived vesicles can modulate the TME by reprogramming immunosuppressive M2-like macrophages toward a pro-inflammatory phenotype (Figure 1). These immunostimulatory vesicles provide a stable, cell-free mechanism, providing effective targeting, modulation, and control of the TME.

A diagrammatic representation of how M1-like macrophage-derived EVs reprogram a tumor-supportive, immunosuppressive microenvironment into an anti-tumor, immunostimulatory one. In the initial state, pro-tumor M2 macrophages support tumor survival, promote angiogenesis, and inhibit killer CD8+ T cells. After introducing M1-like macrophage-derived EVs, existing M2-like macrophages are reprogrammed into active anti-tumor M1-phenotypes, which promote direct tumor cell killing via ROS, NO, and inflammatory cytokines while activating CD8+ T cell cytotoxic responses to attack the tumor [8].

## 4. Overview on Natural Extracellular Vesicles (EVs) and Generation of Artificial Nanovesicles (ANVs)

EVs are nanoscale, membrane-bound particles secreted by cells into the extracellular space that mediate intercellular communication, transferring a diverse cargo of proteins, lipids, and nucleic acids between cells [71]. Their biocompatibility, low immunogenicity, and capacity to penetrate biological barriers, including the tumor stroma, provide significant advantages over conventional synthetic drug delivery systems [72]. In the context of cancer immunotherapy, EVs derived from immune-polarized cells such as M1-like macrophages carry functionally relevant cargo that can modulate the TME, making them potential therapeutic vehicles [73]. However, the clinical translation of natural EVs remains limited by low, variable production yields, innate cargo heterogeneity across vesicle populations, and challenges in large-scale manufacturing [74]. Such shortcomings have driven the development of ANVs, which have the structural and functional attributes of natural EVs and also provide greater control over composition, scalability, and surface functionalization [8]. It is important to note, however, that while ANV engineering principles are well established in general nanomedicine, their specific application to M1 macrophage-derived systems remains a nascent and actively evolving area. The following sections discuss natural EV isolation strategies (Section 4.1) and ANV generation methodologies (Section 4.2), with focus on the extent to which each has been applied specifically in M1 macrophage contexts.

### 4.1. Isolation of Natural Extracellular Vesicles

The isolation of natural EVs is a crucial process where the choice of method directly influences their purity, yield and functional integrity. Due to the heterogeneity of EV populations and the complexity of biological fluids, the ideal technique must be selected based on the specific requirements of the downstream application [8].

#### 4.1.1. Differential Ultracentrifugation and Density-Based Separation

Differential ultracentrifugation remains the most widely used technique for EV isolation, which relies on sequential centrifugation steps at increasing speeds to remove cells, debris, and larger vesicles, pelleting smaller EVs such as exosomes at ultrahigh speeds (>100,000× *g*) [75]. Density gradient centrifugation, typically using sucrose or iodixanol gradients, can be utilized to further enhance purity by separating vesicles based on buoyant density [76]. Although such approaches are effective in isolating EV populations, they are often associated with several limitations, such as co-isolation of protein aggregates, time-intensive protocols, and potential vesicle deformation due to high centrifugal forces [77].

#### 4.1.2. Size-Based Isolation Techniques

Size exclusion chromatography (SEC) and ultrafiltration utilize the nanoscale size range of EVs to achieve separation from soluble proteins and other contaminants [78]. SEC is particularly highlighted for its ability to maintain the native state, as well as structural integrity and biological activity, and provide relatively high purity [79]. However, size-based methods are challenged by limited resolution between vesicle subtypes and can result in diluted EV samples, therefore requiring further concentration steps.

#### 4.1.3. Immunoaffinity-Based Techniques

Such techniques use antibodies against EV surface markers (such as tetraspanins like CD63, CD81, and CD9) to selectively isolate specific vesicle populations [80]. These methods offer high specificity and are particularly useful for isolating EVs from defined cellular origins [81]. Despite their precision, immunoaffinity approaches are limited by low yield, high cost, and potential bias toward specific EV subpopulations, limiting their scalability for clinical applications [82].

#### 4.1.4. Microfluidic and Hybrid Approaches

Recent advances in microfluidics have enabled the development of platforms that include size, charge, and immunoaffinity-based separation with miniaturized systems [83]. Such technologies provide rapid processing, reduced sample requirements, and improved reproducibility. Moreover, hybrid approaches combining multiple isolation principles are also utilized to balance yield and purity [84].

A major challenge in EV isolation is the lack of standardized protocols, which leads to variability in vesicle composition and functional outcomes across studies. Contamination with non-vesicular components and batch-to-batch heterogeneity further complicate clinical translation. Such limitations highlight the need for scalable, reproducible isolation strategies.

### 4.2. Isolation of M1 Macrophage-Derived Extracellular Vesicles

The isolation of EVs from M1-polarized macrophages follows the general methodological framework established for natural EVs, but introduces a set of donor cell-specific considerations that are crucial for preserving phenotypic fidelity and ensuring functional relevance of the isolated vesicles. In accordance with the MISEV2023 guidelines published by the International Society for Extracellular Vesicles, EVs should be characterized using operational terms reflecting their physical characteristics, biochemical composition, and cellular origin, with biogenesis-based terminology such as ‘exosomes’ or ‘microvesicles’ discouraged in favor of size-based or marker-based descriptors [85,86].

M1 polarization is canonically induced by co-stimulation with LPS and IFN-γ, which, together, activate TLR4-NF-κB and JAK-STAT1 signaling cascades, driving the upregulation of pro-inflammatory surface markers, including CD80, CD86, and MHC-II [87,88]. The polarization stimulus itself significantly influences both the quantity and molecular composition of secreted EVs, a consideration with direct implications for isolation yield and downstream functional activity. M1-activated macrophages release EVs at markedly higher levels compared to naïve M0 macrophages, with EV secretion substantially enhanced in the pathological M1 state relative to physiological shedding conditions. Furthermore, M1-like macrophage-derived EVs exhibit a more uniform and smaller size distribution compared to M0-like macrophage-derived EVs, with nanoparticle tracking analysis (NTA) exhibiting a mean diameter of approximately 126 nm and a single concentration peak at approximately 97 nm, suggesting that M1 polarization drives a more homogenous vesicle population. M1-like macrophage–EV samples have been consistently observed to exhibit higher particle concentrations compared to M0 and M2-like macrophage–EV samples, further supporting the idea that M1 macrophages secrete EVs at a greater rate [89].

Prior to conditioned medium collection, donor macrophages must be cultured in EV-depleted medium, typically prepared by ultracentrifugation of FBS at 100,000× *g* for 16–18 h or by use of serum-free formulations, to prevent contamination of the EV fraction with bovine-derived vesicles that would counter downstream functional and proteomic analyses [90]. Following polarization, the conditioned medium is collected and subjected to sequential differential centrifugation: an initial low-speed spin at 300× *g* for 10 min removes intact cells, followed by 2000× *g* for 15 min to pellet cell debris and apoptotic bodies, and 5000× *g* for 15 min to remove large membrane fragments, before a final ultracentrifugation step to pellet the EV fraction. The EV pellet is then obtained by ultracentrifugation at 20,000× *g* for 90 min at 4 °C, with all steps performed at 4 °C to minimize vesicle degradation and membrane destabilization [8].

Confirmation of successful M1-like macrophage-derived EV isolation requires a dual layer of characterization: standard EV identity validation and M1 phenotype-specific verification. Standard EV markers, including the tetraspanins CD63 and CD81, the ESCRT-associated protein TSG101, and the membrane scaffold ALIX, should be confirmed by Western blotting, while negative markers such as calnexin and GAPDH must be absent to exclude contamination with endoplasmic reticulum and cytosolic proteins, respectively [86]. Morphological verification by transmission electron microscopy (TEM) and particle size and concentration analysis by nanoparticle tracking analysis (NTA) are standard characterization steps that, in the case of M1-like macrophage-derived EVs, confirm the expected spherical, double-membrane morphology and sub-200 nm size distribution. M1 phenotypic identity of the isolated EVs should additionally be confirmed by flow cytometric or immunoblot detection of M1-like macrophages associated surface markers such as CD80 and CD86, as well as functional validation of cargo activity; for instance, the presence of pro-inflammatory miRNAs or the capacity to activate NF-κB signaling in recipient cells [91]. EV cargo loading in M1-like macrophages is a highly regulated and dynamic process, with M1-polarized macrophages selectively enriching specific anti-tumor miRNAs in their EVs compared to M0 or M2-like macrophages, underscoring the importance of cargo profiling as a functional potency readout rather than an optional characterization step [8].

A persistent challenge specific to the M1-like macrophage context is the limited yield obtainable from primary cell sources, particularly bone marrow-derived macrophages (BMDMs) or monocyte-derived macrophages from peripheral blood, whose numbers are constrained by donor biology and culture capacity. Ultracentrifugation yield is further restricted by rotor specifications and tube capacities, and the culture duration required to generate sufficient BMDMs for EV extraction prolongs the overall isolation process. To address scalability without compromising M1 phenotypic stability, bioreactor-based macrophage expansion and hollow-fiber filtration systems represent emerging strategies warranting further optimization. The establishment of standardized, MISEV2023-compliant isolation protocols specifically validated for M1-like macrophage-derived EVs, including defined polarization conditions, EV-depleted culture media, sequential centrifugation parameters, and both identity and phenotypic characterization panels, is an essential requirement for the reproducible generation of functionally defined M1-like macrophage-derived EV preparations suitable for preclinical and, ultimately, clinical investigation [92].

### 4.3. Generation of Artificial Nanovesicles (ANVs)

ANVs have been developed as engineered systems that are designed to mimic the structural and functional properties of EVs, along with greater control over composition, scalability, and therapeutic payload [8].

Top-down strategies are a method that involves the physical disruption of parent cells to generate vesicles that retain membrane components and surface proteins characteristic of the source cell [93]. Common techniques for producing these vesicles include serial extrusion, where cells are forced through membranes with defined pore sizes to produce vesicles of controlled dimensions; sonication, which utilizes acoustic energy to disrupt cellular membranes, leading to vesicle reassembly; and nitrogen cavitation, which uses rapid pressure changes to induce membrane fragmentation and vesicle formation. These approaches produce vesicles that resemble natural EVs in terms of membrane composition and biological functions, including retention of targeting ligands and immune-modulatory molecules [94,95,96].

Bottom-up strategies produce artificial vesicles by assembling lipids, polymers, or hybrid materials. Liposomes and polymeric nanoparticles can be functionalized with proteins, peptides, or membrane fragments to mimic EV-like properties [97]. Biomimetic nanovesicles can also be designed by coating synthetic nanoparticles with cell-derived membranes, thereby combining the tunability of synthetic systems with the biological identity of natural membranes [98]. This allows precise control over vesicle size, composition, and surface functionality while preserving targeting capabilities.

Compared to natural EVs, ANVs offer translational advantages that address the core limitations of natural vesicles. One of the major shortcomings that ANVs overcome is scalability: ANVs can be produced at higher, reproducible yields through cell extrusion, sonication, or microfluidic fabrication methods, making them compliant with the manufacturing demands of clinical application [99]. Another advantage is the compositional control they afford: therapeutic cargo, surface-targeting ligands, and membrane components can be utilized in a tunable manner, providing precise customization of vesicle function [8]. This engineering flexibility extends to modifications that enhance physicochemical stability, optimize pharmacokinetic profiles, and program stimuli-responsive drug release kinetics. Furthermore, ANVs exhibit reduced population heterogeneity relative to natural EVs, yielding more uniform size distributions and cargo profiles that are required for regulatory compliance and inter-batch reproducibility [8]. Collectively, these properties position ANVs as better platforms for targeted drug delivery, where consistency, predictability, and scalability are critical. Despite their advantages, ANVs may lack certain complex biological features that are characteristics of natural EVs, such as specific cargo sorting mechanisms, which are regulated by the Endosomal Sorting Complexes Required for Transport (ESCRT) machinery, lipid raft microdomains, and specific RNA-binding proteins and dynamic responsiveness to environmental factors [100]. Recent studies have demonstrated the therapeutic potential of M1-like macrophage-derived EVs and ANVs across multiple cancer models, employing diverse cargoes such as chemotherapeutic agents, miRNAs, and photosensitizers (Table 1).

### 4.4. M1-like Macrophage-Derived ANVs: Current Evidence and Limitations

Despite the conceptual promise of M1-like macrophage-derived ANVs, research studies based on this system remain nascent. The majority of published ANV studies utilize non-immune or generic macrophage cell sources, with relatively few reports utilizing M1-polarized macrophages as the donor cell population for engineered vesicle fabrication. General ANV engineering principles, including top-down extrusion, bottom-up liposomal assembly, and hybrid membrane-coating strategies, are therefore being extrapolated to M1-like macrophage contexts rather than derived from an extensive M1-like macrophage-specific evidence base. Among the limited but informative exceptions, nanovesicles generated by serial extrusion of M1-polarized macrophages have demonstrated retention of surface immunostimulatory molecules and M1-associated cargo, enabling tumor-associated macrophage repolarization and synergistic immune checkpoint inhibition [101]. Similarly, M1-like macrophage membrane-coated liposomes incorporating doxorubicin and a triosephosphate isomerase inhibitor demonstrated synergistic tumor suppression in osteosarcoma models with reduced systemic toxicity [107], and M1-like macrophages nanovesicle-coated lipid nanoparticles co-delivering Bcl2-siRNA (small interfering RNA) with cytokines demonstrated potent colon cancer suppression via NK and T cell activation [111]. Most recently, it was also demonstrated that M1-like macrophage-derived small EVs exhibit intrinsic anticancer activity by evading immune surveillance and suppressing cancer growth through endogenous enrichment of antiproliferative miRNAs, and that this activity can be further augmented by exogenous drug loading for a synergistic therapeutic effect [113]. Collectively, this growing but still limited body of evidence represents a significant opportunity for future research studies. Recent studies have demonstrated the broader therapeutic potential of M1-like macrophage-derived EVs and ANVs across multiple cancer models, using diverse cargoes such as chemotherapeutic agents, miRNAs, and photosensitizers, as summarized in Table 1.

## 5. Cargo Loading

Effective incorporation of therapeutic payloads into EVs and ANVs is crucial for determining their utility as drug delivery platforms. Cargo loading strategies must consider vital factors such as encapsulation efficiency, vesicle integrity, and functional preservation of both cargo and carrier. These approaches are broadly classified into passive and active loading techniques, each with distinct advantages and limitations based on the physicochemical properties of the cargo and the intended application [8].

### 5.1. Passive vs. Active Loading Techniques

#### 5.1.1. Passive Loading Strategies

Passive loading is a non-disruptive method that depends on spontaneous processes, such as diffusion, hydrophobic interactions, and electrostatic forces, to incorporate therapeutic agents into vesicles [114]. This approach can be used either during vesicle biogenesis (in donor cells) or through post-isolation incubation [8,114]. A commonly used strategy involves pre-loading donor cells, where cells are exposed to a therapeutic agent, which is subsequently incorporated into EVs during natural vesicle formation. This technique is particularly effective for nucleic acids and small molecules which can be endogenously packaged through cellular sorting mechanisms [114,115]. Alternatively, co-incubation of isolated vesicles with cargo allows for diffusion-based loading, especially for lipophilic drugs that can integrate into the vesicle membrane [116].

Major advantages of passive loading include minimal disruption to vesicle structure and preservation of membrane proteins. Although passive loading is simple to operate, it is often limited by low encapsulation efficiency, lack of control over cargo distribution, and dependence on the intrinsic loading capacity of vesicles [8]. Moreover, variability in cellular uptake and intracellular trafficking can cause substantial inconsistency in the final cargo amount [117,118].

#### 5.1.2. Active Loading Strategies

Active loading uses external physical or chemical forces to transiently disrupt vesicle membranes, which allows for direct incorporation of therapeutic cargo [8]. Such methods provide higher encapsulation efficiency but may compromise vesicle integrity if not carefully optimized [119]. Some of the common active loading approaches include electroporation, sonication, freeze–thaw cycles, chemical transfection and membrane permeabilization, and extrusion-based loading [120]. In electroporation, an electric field is applied to induce temporary pore formation in vesicle membranes, which enables the entry of charged molecules such as siRNA, miRNA, mRNA and plasmid DNA. Although commonly used, electroporation can cause cargo aggregation and vesicle destabilization under suboptimal conditions [121]. Through sonication, the ultrasonic waves generate mechanical shear forces that disrupt membrane structure, which allows the cargo to be incorporated during membrane reassembly. This technique is particularly effective for loading hydrophobic drugs but may change the membrane protein orientation [122]. Membrane permeability is also induced by repeated freezing and thawing, which enables cargo entry through ice crystal formation. However, this method can lead to vesicle fusion and reduced homogeneity [123]. In the chemical transfection method, cargo loading can be enhanced by using agents such as saponin or surfactants, which can transiently increase membrane permeability. However, care must be taken to avoid residual toxicity or irreversible membrane damage [124,125]. In ANVs, cargo can be incorporated during vesicle formation by co-extruding cellular material or synthetic components with the therapeutic payload, which ensures a uniform encapsulation [126].

Active loading methods ensure greater control over cargo quantity and are particularly suitable for large or hydrophilic molecules [127]. However, they require careful optimization to balance loading efficiency with preservation of vesicle functionality, especially when surface proteins are critical for targeting.

### 5.2. Cargo Loading Strategies Specific to M1-like Macrophage-Derived EVs

The cargo loading of M1-like macrophage-derived EVs is determined by the unique physicochemical properties of the vesicle membrane and the nature of the intended therapeutic payload, and the choice of loading method carries direct consequences for both encapsulation efficiency and preservation of M1-like macrophage-associated surface identity. For hydrophobic small-molecule chemotherapeutics, sonication has emerged as the method of choice. When paclitaxel was loaded into M1-like macrophage-derived exosomes via sonication, an encapsulation efficiency of 19.55 ± 2.48% was achieved, substantially outperforming simple co-incubation (4.85 ± 1.65%), with the size increase of the loaded vesicles attributed to cargo intercalation into the lipid bilayer without alteration of surface marker expression [8]. This principle was extended to docetaxel, where sonication-based loading into M1-like macrophage-derived exosomes produced a drug delivery system capable of maintaining durable M1-like macrophage activation in the TME and resisting IL-4-driven repolarization to M2-phenotype, an advantage attributed to drug-induced mitochondrial reprogramming [128]. Similarly, gemcitabine has been loaded into M1-like macrophage-derived exosomes via ultrasonication, yielding a synergistic chemoimmunotherapeutic system with enhanced anti-bladder cancer activity compared to free drug [129]. For hydrophilic and macromolecular cargo, including cisplatin and nucleic acid-based therapeutics, active loading techniques are required. M1-like macrophage-derived exosomes loaded with cisplatin via co-incubation under mildly acidic conditions demonstrated preferential tumor accumulation and improved anti-tumor efficacy in lung cancer models, consistent with the enhanced permeability afforded by the M1-like macrophage-derived EV surface profile [130]. Across these chemotherapeutic loading studies, a critical shared finding is that sonication achieves markedly superior encapsulation efficiency over passive incubation or electroporation for hydrophobic drugs in M1-like macrophage-derived EV systems, with one comparative analysis in macrophage-derived exosomes reporting sonication loading capacity of 28.3% versus 5.3% by electroporation and 1.4% by incubation [8].

For nucleic acid cargo, including miRNAs and siRNAs, M1-like macrophage-derived EVs benefit from two complementary loading strategies: endogenous enrichment through donor cell manipulation, and exogenous electroporation. M1-polarized macrophages naturally enrich specific anti-tumor miRNAs in their secreted EVs as part of their inflammatory phenotype; miR-155, for instance, is selectively upregulated and packaged into M1-like macrophage-derived exosomes, where it functions to suppress oncogenic signaling pathways in recipient cells, making endogenous cargo an intrinsic therapeutic asset of this platform [131]. To extend this endogenous cargo with exogenously defined nucleic acid payloads, electroporation is the predominant strategy, as the transient membrane poration it induces enables entry of larger nucleic acid species, including siRNA and miRNA mimics, that cannot passively diffuse across the lipid bilayer [132]. The specificity of electroporation for particular EV subpopulations can be further refined: magnetic bead-based selection of surface marker-defined EV populations prior to electroporation has been shown to improve loading efficiency and reduce non-specific cargo association, a refinement particularly relevant for M1-like macrophage-derived EVs where CD80 or CD81 surface markers can be exploited for subpopulation enrichment [133].

**Figure 2:**
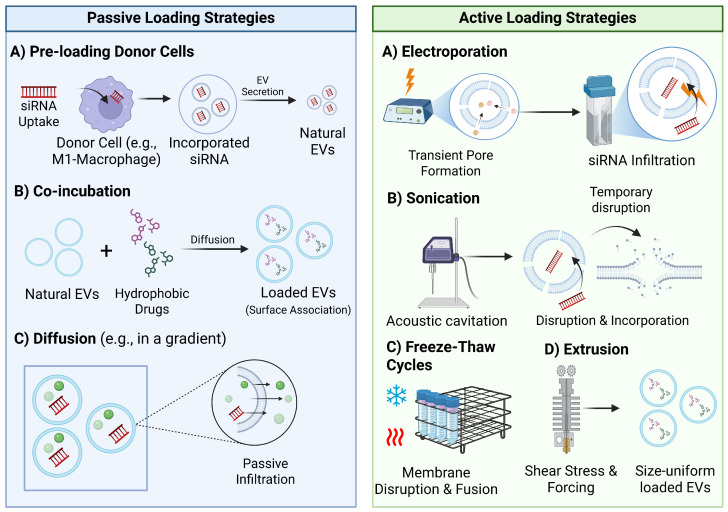
Illustration of various active and passive loading strategies of cargo in extracellular vesicles (EVs). Passive (pre-loading, co-incubation, diffusion) and active (electroporation, sonication, freeze–thaw, extrusion) strategies for loading cargos into EVs. EVs: extracellular vesicles; siRNA: small interfering RNA. Created in BioRender. Subramaniyan, S. (2026) https://BioRender.com/kf9vuxb (accessed on 24 May 2026).

Regardless of loading method, it is essential to verify that the loading process does not compromise the M1 phenotypic identity of the vesicle, particularly the retention of pro-inflammatory surface markers such as CD80 and CD86, as disruption of these molecules would undermine the dual cargo-delivery and immunomodulatory function that distinguishes M1-like macrophage-derived EVs from generic nanocarrier systems. Various active and passive loading strategies of cargo are summarized in Figure 2.

### 5.3. Stability and Efficiency of Cargo Encapsulation

The therapeutic efficacy of vesicle-based delivery systems is closely linked to the stability of the encapsulated cargo and the efficiency of encapsulation, both of which influence biodistribution, release kinetics, and target specificity [134]. The efficiency of encapsulation varies considerably depending on the loading method, physicochemical properties of the cargo, and compositional characteristics of the vesicle membrane [135]. Hydrophobic small molecules achieve higher loading efficiencies because of their thermodynamic affinity for lipid bilayers, whereas hydrophilic compounds and larger biomolecules, including nucleic acids and proteins, typically require active loading strategies for the same [136]. Beyond cargo properties, encapsulation efficiency is further modulated by membrane fluidity and composition, which determine bilayer permeability and extrinsic loading conditions, including temperature, ionic strength, and pH [137]. ANVs offer a distinct advantage in this context, as their defined lipid compositions allow for more controlled and reproducible loading compared to the heterogeneous membranes of natural EVs [138]. Quantitative assessment of encapsulation efficiency, expressed as the ratio of encapsulated cargo to total input cargo, is therefore an essential parameter in the characterization and optimization of any vesicle-based formulation [139].

Maintaining cargo stability during storage and after systemic administration remains a major challenge in vesicle-based drug delivery [140]. Encapsulated cargo must be protected from enzymatic degradation, premature leakage, and environmental challenges such as pH fluctuations and oxidative conditions [141]. Both EVs and ANVs provide a protective lipid bilayer that protects sensitive molecules, such as RNAs and proteins, from degradation. However, cargo leakage may occur due to membrane instability, particularly under physiological shear stress or during long-term storage [142]. Various strategies can be used to overcome such challenges and improve cargo stability: optimization of lipid composition to enhance membrane rigidity; surface modification approaches, such as PEGylation, to reduce aggregation and clearance; and biopreservation techniques, including lyophilization and cryopreservation for long-term storage [8,143,144,145]. It is crucial to balance high encapsulation efficiency and preservation of vesicle functionality in cargo loading. Harsh loading methods may increase cargo incorporation but can disrupt membrane proteins responsible for targeting and cellular uptake, and conversely, gentler methods may yield suboptimal drug loading despite preserving biological function [146]. In this context, ANVs enable precise control over composition and loading conditions, thereby allowing the optimization of both efficiency and stability [143]. Nonetheless, achieving an optimal balance remains a key challenge in developing vesicle-based drug delivery systems.

## 6. Cargo Delivery

The therapeutic outcome of EVs and artificial ANVs as drug delivery platforms depends on their ability to selectively target tumor tissues, achieve efficient cellular uptake, and ensure functional release of cargo within recipient cells. These processes are controlled by a combination of vesicle properties and engineered modifications that influence biodistribution, cellular uptake, and intracellular trafficking.

### 6.1. Targeting Strategies: Ligand Modification and Tropism

A key advantage of macrophage-derived EVs is their biological tropism toward inflamed and tumor tissues. This targeting capability is mediated by the expression of adhesion molecules, chemokine receptors, and integrins on the vesicle surface, which allows the interactions with endothelial cells and tumor-associated cells [147]. Vesicles derived from M1-like macrophages show enhanced affinity for inflammatory microenvironments, enabling preferential accumulation within tumors [148]. This ability provides a foundation for targeted delivery without the need for extensive surface modification.

To further improve specificity and targeting efficiency, vesicles can be modified through surface functionalization with targeting ligands. These include antibodies/antibody fragments targeting tumor-associated antigens (for example, HER2, EGFR), peptides that recognize specific receptors overexpressed on cancer cells, aptamers that provide high-affinity nucleic acid-based targeting, and small molecules like folate that exploit receptor-mediated uptake pathways [149,150]. These ligands can be introduced by genetically modifying the donor cells (for EVs) or through chemical conjugation and insertion (for ANVs) [8]. Such modifications significantly enhance binding specificity, cellular uptake, and therapeutic efficacy. Advanced targeting strategies utilize unique characteristics of the TME, including acidic pH, hypoxia, and enzyme-rich conditions. Vesicles can be modified to exhibit stimuli-responsive behavior, such as pH-sensitive membrane destabilization for enhanced drug release in acidic tumor regions and enzyme-cleaved linkers that activate targeting ligands in the presence of tumor-associated proteases [151]. These approaches enable spatiotemporal control of drug delivery, reducing off-target effects while improving therapeutic impact.

Even with such advances, achieving precise targeting remains challenging due to factors such as off-target uptake by the mononuclear phagocyte system (MPS), variability in receptor expression across tumors, and biological barriers such as the extracellular matrix [8]. Furthermore, optimizing targeting strategies requires a balance between specificity, circulation time, and immune evasion.

### 6.2. Mechanisms of Cellular Uptake and Intracellular Trafficking

Following successful targeting, vesicles must be internalized by recipient cells to deliver their cargo. EVs and ANVs can enter cells through multiple endocytic and non-endocytic pathways, such as clathrin-mediated endocytosis (receptor-dependent process that enables the uptake of ligand-functionalized vesicles), caveolin-mediated endocytosis (a lipid-raft-dependent process involving the inward budding of 50–80 nm flask-shaped, cholesterol-rich plasma membrane invaginations called caveolae), macropinocytosis (non-specific uptake mechanism involving membrane ruffling and vesicle engulfment), phagocytosis (cells engulf large particles (0.5 μm), particularly relevant for immune cells), and direct membrane fusion (vesicles fuse with the plasma membrane, releasing cargo directly into the cytosol) [152,153,154]. The predominant uptake mechanism depends on vesicle size, surface composition, and the recipient cell type.

Once internalized, vesicles are typically transported through the endosomal-lysosomal pathway. Early endosomes mature into late endosomes and eventually fuse with lysosomes, where acidic and enzymatic conditions can lead to cargo degradation. This is a major barrier to effective intracellular delivery, especially for sensitive molecules such as RNAs [155]. Vesicles must either escape from endosomes before lysosomal degradation or resist degradation and release cargo in a controlled manner to achieve functional delivery [156]. Endosomal escape can be enhanced by strategies such as membrane destabilization, incorporation of fusogenic lipids, and pH-responsive materials that disrupt endosomal membranes [157].

Vesicle-mediated cargo delivery is successful if the therapeutic cargo reaches its intracellular target in an active form. For example, siRNA and miRNA must access the cytoplasm to engage RNA interference pathways, or small molecule drugs must be released at sufficient concentrations to produce cytotoxic effects [8,158]. The efficiency of cytosolic release is crucial for determining therapeutic outcome. Several biological barriers limit the efficiency of vesicle-mediated cargo delivery, including endosomal entrapment, lysosomal degradation, intracellular sequestration, and limited diffusion within the cytoplasm [159]. Overcoming these barriers remains a major focus in designing next-generation vesicle systems. M1-like macrophage-derived vesicles provide a unique advantage in cargo delivery by combining targeting capability with immunomodulatory activity. Upon uptake by tumor or immune cells, these vesicles can simultaneously deliver therapeutic agents and induce pro-inflammatory signaling, thereby enhancing anti-tumor responses [160]. This functionality differentiates M1-like macrophage-derived EVs and ANVs from conventional nanoparticle systems and highlights their potential as multifunctional therapeutic platforms.

### 6.3. M1-like Macrophage-Derived EVs for Cargo Delivery

A distinguishing feature of M1-like macrophage-derived EVs, relative to generic synthetic nanocarriers, is the presence of an intrinsic biological targeting apparatus inherited from the parent cell. The surface proteome of M1-like macrophage-derived small EVs has been shown to include a range of TME-recruiting proteins, including β2 integrin, galectin-3 (Gal-3), VEGFR1, CD44, and neuropilin-1 (NRP1), that collectively mediate adhesion to extracellular matrix components, interactions with tumor vasculature, and enhanced penetration into solid tumor masses [161]. M1-like macrophage-derived EVs have also been shown to express CD47 on their surface, the canonical ‘don’t eat me’ signal that engages SIRPα on myeloid cells to suppress phagocytic clearance, thereby enabling immune evasion and prolonging systemic circulation, a property that significantly enhances in vivo tumor accumulation [162]. This combination of active tumor-homing surface ligands and passive immune-evasive signaling affords M1-like macrophage-derived EVs a dual targeting advantage that is fundamentally distinct from the non-specific accumulation mechanisms of conventional lipid nanoparticles. Beyond their intrinsic tropism, M1-like macrophage-derived EVs have demonstrated the capacity to cross formidable biological barriers: M1-like macrophage-derived EVs administered intravenously achieved blood–brain barrier penetration in a glioblastoma model, accumulating within the tumor microenvironment and driving M2-to-M1 phenotypic tumor-associated macrophage (TAM) repolarization, a finding attributed to the macrophage-derived membrane’s native transcytosis competence [163]. This barrier-crossing capability, combined with the inflammatory homing properties provided by M1 polarization, positions M1-like macrophage-derived EVs as particularly well-suited for tumors situated within immunologically restricted anatomical compartments.

Upon arrival at tumor tissue, M1-like macrophage-derived EVs engage recipient cells, including tumor cells, TAMs, and stromal cells, through multiple internalization pathways, with the predominant route determined by vesicle size, surface composition, and recipient cell identity [155,164]. Clathrin-mediated endocytosis is the principal pathway for receptor-ligand-dependent uptake, wherein surface proteins such as integrins and CD44 on M1-like macrophage-EVs engage cognate receptors on tumor cells to initiate internalization [164]. In the context of TAM re-education, a therapeutically critical application of M1 EVs, macropinocytosis and phagocytosis are particularly relevant, as these are high-capacity uptake mechanisms native to macrophage biology that facilitate bulk internalization of EVs and their immunostimulatory cargo [155]. Following internalization, cargo derived from M1-like macrophage-derived EVs enters the canonical endosomal-lysosomal trafficking pathway; however, a subset of cargo, particularly miRNAs such as miR-155, miR-150, and miR-34a that are endogenously enriched in M1-like macrophage-EVs, achieves cytoplasmic access through back-fusion of the EV membrane with the endosomal limiting membrane, enabling engagement of the RNA interference machinery without requiring active endosomal escape strategies [8,165].

**Figure 3:**
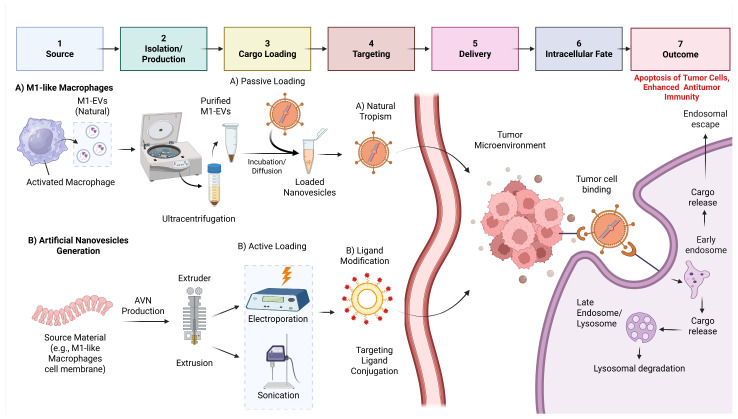
Functional workflow of EV/ANV-based drug delivery systems. Isolation/production, cargo loading, targeting (tropism/ligands), delivery to tumors, cellular uptake, intracellular trafficking, and cargo release leading to tumor apoptosis and enhanced anti-tumor immunity. M1: M1-polarized macrophages; EVs: extracellular vesicles; M1-EVs: M1-polarized macrophage-derived extracellular vesicles; AVNs: artificial nanovesicles; Cargo: therapeutic or bioactive payload; Ligand: targeting moiety conjugated to vesicle surface; Tumor Microenvironment: local cellular environment surrounding tumor; Endosome: intracellular vesicular compartment; Lysosome: degradative organelle involved in cargo processing. Created in BioRender. Subramaniyan, S. (2026) https://BioRender.com/ti2qd1v (accessed on 24 May 2026).

Upon cytoplasmic delivery, M1-like macrophage-EV cargo simultaneously exerts direct anti-proliferative effects on tumor cells and activates NF-κB-dependent pro-inflammatory signaling in recipient immune cells, as demonstrated by the finding that M1-like macrophage-derived EV uptake by naïve M0 macrophages induces robust M1 repolarization through TLR4-NF-κB pathway activation [88]. This dual intracellular signaling outcome, cytotoxic cargo activity in tumor cells alongside immunostimulatory reprogramming of immune cells, represents the mechanistic basis of the multifunctional therapeutic identity of M1-like macrophage-derived EVs. The overall workflow of EV/ANV-based drug delivery, from vesicle generation to cargo delivery and intracellular release, is summarized in Figure 3.

Figure 3 demonstrates the development of macrophage-derived drug delivery platforms, showing the engineering of both natural M1-polarized EVs and ANVs. Following isolation via ultracentrifugation or extrusion, these vesicles are loaded with therapeutic cargo, such as anti-cancer drugs, using passive incubation or active loading methods like electroporation and sonication. Surface modification with targeting ligands ensures specific binding and delivery to tumor tissue, where the loaded nanovesicles are internalized, escape endosomal degradation, and release their cargo to induce tumor cell death and enhance anti-tumor immune responses [166].

### 6.4. Biodistribution, Safety, and Off-Target Considerations for M1-like Macrophage-Derived EVs

A critical and clinically relevant dimension of M1-like macrophage-derived EV therapy is the relationship between administration route, biodistribution, and the safety profile arising from their pro-inflammatory molecular cargo. Unlike conventional nanoparticles carrying cytotoxic payloads, M1-like macrophage-derived EVs are inherently bioactive carriers whose membrane-associated and luminal content, including TNF-α, IL-1β, IL-6, iNOS-derived signaling intermediates, and immunostimulatory miRNAs such as miR-155, can activate innate immune signaling cascades in off-target tissues independently of any exogenously loaded drug [167]. Understanding how the administration route regulates the initial biodistribution of these vesicles is therefore a prerequisite for translating their therapeutic potential without inducing systemic inflammatory toxicity.

Following intravenous administration, the most commonly employed route in preclinical M1-like macrophage-derived EV studies, vesicles encounter the MPS as their primary clearance mechanism, with hepatic Kupffer cells and splenic macrophages rapidly sequestering circulating EVs within minutes of injection [8]. While macrophage-derived EVs benefit from surface expression of CD47, the signal that attenuates SIRPα-mediated phagocytic clearance and prolongs circulation time relative to synthetic nanoparticles, this protection is partial and does not fully prevent hepatosplenic accumulation [162,168]. M1-like macrophage-derived EV accumulation in the liver and spleen is not merely a pharmacokinetic inefficiency; it carries a direct safety implication, as the delivery of pro-inflammatory M1-like macrophage cargo to hepatic macrophages or splenic immune cells could potentiate local inflammatory responses in these organs, particularly under repeated dosing regimens [169]. Alternative administration routes, including intratumoral injection, which bypasses systemic MPS exposure entirely, and locoregional delivery strategies, have demonstrated improved tumor accumulation and reduced off-target distribution in preclinical models, and merit consideration particularly for anatomically accessible tumors [170].

The cell source used for M1-like macrophage-derived EV production carries its own biodistribution and safety implications. EVs generated from immortalized macrophage cell lines such as RAW264.7 and THP-1 exhibit different surface glycoprotein profiles compared to primary bone marrow-derived or monocyte-derived macrophages, which affects their receptor-mediated interactions with endothelial cells and immune surveillance mechanisms in vivo [171]. Allogeneic cell sources introduce the additional risk of immune recognition of donor-specific surface antigens on the EV membrane, potentially triggering adaptive immune responses upon repeated administration, a concern that autologous patient-derived macrophage EV strategies are specifically designed to circumvent [172]. Furthermore, the degree of M1 polarization achieved in donor cells, which varies with polarization protocol, stimulation duration, and passage number, directly determines the inflammatory potency of the resultant EVs, making polarization status a key safety variable that must be rigorously controlled and documented in any translational program [8].

Vesicle engineering strategies offer a rational approach to improving the safety-efficacy balance of M1-like macrophage-derived EV therapy. Surface PEGylation attenuates MPS recognition and extends circulation half-life, improving tumor accumulation while simultaneously reducing hepatosplenic off-target deposition [173]. Stimuli-responsive masking strategies, including pH-sensitive surface coatings that shield pro-inflammatory surface proteins at physiological pH but release them selectively within the acidic TME, represent an emerging approach to spatially confine M1 immunostimulatory activity to tumor tissue, preventing systemic cytokine signaling [58]. For hybrid M1 EV-liposome platforms, encapsulation of the M1 membrane within a PEGylated lipid shell has been shown to reduce premature immune activation during systemic circulation while preserving intratumoral immunostimulatory capacity upon lipid shell degradation [174]. These engineering strategies demonstrate that the safety and biodistribution profile of M1 macrophage-derived EVs is not fixed but can be actively modulated through rational surface design, provided that functional M1 identity is verified to be preserved post-modification, which remains a non-trivial characterization challenge [175].

## 7. Therapeutic Applications of M1-like Macrophage-Derived EVs and ANVs in Cancer

M1-like macrophage-derived EVs have shown broad preclinical therapeutic activity spanning multiple cancer types, operating through two principal and complementary mechanisms: direct cytotoxic cargo delivery and immunological reprogramming of the tumor microenvironment. A central application has been the TAM-targeted re-education strategy, wherein M1-like macrophage-derived EVs carrying miRNAs and immunostimulatory proteins selectively reprogram immunosuppressive M2-polarized TAMs toward an anti-tumor M1 phenotype. Demonstrating this, it was shown that IL4R-targeted M1-like macrophage-derived exosomes co-loaded with miR-511-3p and NF-κB p50 siRNA selectively reprogrammed IL4R-expressing TAMs in breast and lung cancer models, simultaneously restraining tumor growth and potentiating systemic anti-tumor immunity [102]. Complementing this immunological approach, Li and team showed that M1-like macrophage-derived exosomes delivering miR-16-5p suppressed PD-L1 expression in gastric cancer, restoring T cell-mediated tumor killing [103], while Desai et and team showed that M1-like macrophage-derived ANVs carrying caspase-activating proteins induced caspase 3/7-mediated apoptosis in triple-negative breast cancer cells [112].

A parallel body of work has established M1-like macrophage-derived EVs as effective carriers of conventional chemotherapeutic and photodynamic agents, leveraging their inherent tumor-homing properties and immune-evasive surface profile to improve therapeutic index. Paclitaxel- and docetaxel-loaded M1-like macrophage-derived exosomes demonstrated enhanced anti-tumor activity and improved drug delivery in breast cancer models, respectively [8,104], while cisplatin-encapsulating M1-like macrophage-derived exosomes achieved preferential tumor accumulation and improved regression in ovarian cancer [108]. In the photodynamic space, zinc phthalocyanine-loaded M1-like macrophage-derived exosomes improved photodynamic therapy efficacy in colon cancer [105], and M1-like macrophage-derived exosomes co-delivering the photosensitizer Ce6 alongside endogenous nitric oxide enabled dual photodynamic and gas therapy in orthotopic colorectal cancer [110]. Exosome-mimetic M1-like macrophage-derived ANVs carrying docosahexaenoic acid further induced apoptosis and suppressed hepatocellular carcinoma growth [109].

Hybrid vesicle platforms have introduced additional engineering sophistication to the M1-like macrophage-derived EV therapeutic framework. M1-like macrophage-derived EV-thermoresponsive liposome hybrids enabled controlled drug release and effective tumor suppression in melanoma [8], while M1-like macrophage-derived EV-liposome hybrids co-delivering REV (reversine), SR780Fe (photosensitizer), and the tumor-penetrating RS17 peptide demonstrated enhanced targeting and augmented immunogenic cell death in breast cancer [8]. Collectively, these studies, spanning TAM repolarization, miRNA delivery, chemotherapy, photodynamic therapy, and hybrid engineering, highlight the multifunctional therapeutic versatility of M1-like macrophage-derived vesicle systems across diverse oncological contexts.

Recent studies have demonstrated the therapeutic potential of M1-like macrophage-derived EVs and ANVs across multiple cancer models, employing diverse cargoes such as chemotherapeutic agents, miRNAs, and photosensitizers (Table 1).

## 8. Challenges and Future Perspectives

Despite significant advances in the development of EVs and ANVs as drug delivery platforms, several critical challenges continue to affect their clinical translation. These limitations include issues related to production scalability, standardization, reproducibility, and functional consistency, as well as broader concerns regarding safety, regulatory approval, and patient-specific variability. Addressing such challenges will be essential for realizing the full therapeutic potential of vesicle-based delivery systems in cancer.

### 8.1. Challenges in the Development of M1-like Macrophage-Derived EVs and ANVs

Beyond the general production and standardization challenges that affect all EV-based platforms, M1-like macrophage-derived EVs and ANVs face a distinct set of biological and translational hurdles rooted in the nature of their donor cell. One of the primary limitations of natural EV-based systems is the low yield of vesicle production, particularly when derived from primary cells such as macrophages. The secretion of EVs is limited by cellular physiology, making it difficult to obtain sufficient quantities for clinical applications without extensive cell culture expansion [176]. This not only increases cost and time requirements but also introduces variability associated with prolonged cell culture, including phenotypic drift and varied vesicle composition [169]. In M1-phenotype macrophage systems, this challenge is compounded by the sensitivity of the M1 polarization state itself: the M1-phenotype is not a fixed binary state but an activation profile highly sensitive to cytokine concentrations, stimulation duration, and inter-donor biological differences, all of which directly determine the molecular composition and immunostimulatory potency of the resulting EVs [177]. Variability in M1 polarization protocols across laboratories, including the choice of macrophage source (peripheral blood monocyte-derived, bone marrow-derived, or immortalized cell lines such as RAW264.7 and THP-1), produces EV populations with substantially different surface marker profiles, miRNA cargo, and functional capacity, compounding the already significant reproducibility challenge [8]. So far, there is a lack of universally accepted standards for EV isolation and purification, quantification and characterization, and functional validation assays [178,179]. This absence of standardization leads to significant variability across studies, limiting reproducibility and comparability of results [180,181]. For M1-like macrophage-derived EVs specifically, this gap extends to the absence of defined minimum potency indicators, such as validated co-expression thresholds for CD80/CD86, quantitative miR-155 enrichment, or functional NF-κB activation capacity in recipient macrophages, without which batch-to-batch consistency cannot be meaningfully assessed [8,113]. Ensuring batch-to-batch consistency is critical for clinical translation. Variations in donor cell conditions, culture environments, and loading techniques can influence vesicle composition, including surface markers, lipid profiles, and cargo content. These variations can, in turn, affect targeting efficiency, biodistribution, and therapeutic outcomes [182].

A second class of challenges is specific to the pro-inflammatory nature of M1-like macrophage-derived EV cargo. Unlike immunologically inert nanocarriers, M1-like macrophage-derived EVs carry biologically active pro-inflammatory molecules, including TNF-α, IL-6, IL-1β, and miR-155, that can activate TLR4-NF-κB and related inflammatory pathways in off-target tissues, with the potential to drive systemic inflammation if vesicle biodistribution is not precisely controlled [88]. This immunostimulatory risk is particularly relevant in patients with pre-existing inflammatory conditions. The development of hybrid M1-like macrophage-derived EV platforms introduces an additional layer of complexity: fusing M1-like macrophage-derived membrane material with synthetic liposomes or lipid nanoparticles can unpredictably alter the density, orientation, and accessibility of M1-phenotype-specific surface proteins, potentially diminishing the tumor-homing and immunostimulatory properties that differentiate M1-like macrophage-derived EVs from generic nanocarriers [8]. Characterizing how hybridization affects M1-phenotype functional identity post-fabrication, not merely physicochemical properties, remains an unresolved methodological gap. It was further highlighted that EVs as gene delivery vehicles face distinct immunogenicity profiling requirements and translational hurdles that differ fundamentally from those of lipid nanoparticles and viral vectors, underscoring the need for platform-specific regulatory pathways [183]. For clinical translation, it is essential to establish clear regulatory frameworks that standardize manufacturing protocols, quality control, and therapeutic usage.

### 8.2. Future Directions for Clinical Translation and Personalized Medicine

Addressing the donor variability challenge will require the development of standardized, Good Manufacturing Practice (GMP)-compatible M1 polarization protocols that define precise cytokine concentrations, stimulation windows, and validated release criteria for EV functional potency. A promising near-term strategy is donor cell pooling: combining macrophages from multiple donors prior to polarization and EV collection has been shown to reduce inter-individual phenotypic variability without fundamentally altering macrophage activation state or functional capacity, offering a practical path toward more reproducible EV manufacturing [177]. Future efforts are likely to focus on the development of hybrid vesicle systems that combine the biological advantages of natural EVs with the controllability of synthetic nanomaterials. These may include EV-liposome hybrids for improved loading and stability, membrane-coated nanoparticles that retain cell-specific targeting properties, and stimuli-responsive vesicles capable of controlled, on-demand drug release [184]. For M1-like macrophage-derived EV hybrid platforms specifically, future work must develop standardized functional characterization assays, beyond physicochemical metrics, that validate retention of M1 biological identity post-hybridization, including TAM repolarization capacity and pro-inflammatory cytokine induction in recipient immune cells [8,183].

To mitigate the off-target inflammatory risk inherent to M1-like macrophage-derived EV cargo, future engineering strategies should incorporate tumor-responsive activation mechanisms that confine M1-phenotype immunostimulatory activity to the TME. Recent advanced solutions, such as pH-responsive materials, fusogenic lipids, and stealth coatings, are being developed to improve delivery efficiency and prolong circulation time [185]. When applied specifically to M1-like macrophage-derived EV systems, pH-sensitive membrane masking moieties and MMP-cleavable surface modifications represent emerging approaches to spatiotemporally restrict NF-κB activation to tumor tissue, reducing systemic inflammatory exposure [186]. Integration with omics technologies (genomics, transcriptomics, proteomics) and artificial intelligence-driven design may further allow for the customization of vesicle-based therapies based on tumor-specific profiles. Single-cell transcriptomic profiling of donor macrophage populations combined with EV cargo analysis will enable identification of optimal polarization states that maximize therapeutic miRNA enrichment and surface marker integrity, moving from empirical protocol optimization toward data-driven M1-like macrophage-derived EV design [187]. Patient-derived macrophages can be used to generate autologous EVs, reducing the risk of immunogenicity and enabling tailored therapeutic applications. Additionally, vesicles can be loaded with patient-specific molecular cargo, such as siRNAs targeting individual oncogenic mutations [188]. Collaborative efforts between academia, industry, and regulatory agencies will be critical in advancing vesicle-based therapeutics from bench to bedside.

## 9. Conclusions

The convergence of immunology with nanotechnology has advanced the development of biomimetic drug delivery systems, making macrophage-derived vesicles a promising therapeutic platform. The unique functional attributes of M1-polarized macrophages, including their pro-inflammatory signaling capacity, antigen-presenting ability, and intrinsic tumor-homing properties, provide a strong foundation for designing vesicle-based therapeutics aimed at reprogramming the TME and enhancing multi-layered anti-tumor immunity. EVs provide a biologically compatible and versatile delivery system that can transfer complex immunomodulatory cargo, while ANVs address critical limitations related to scalability, compositional control, and inter-batch reproducibility. Such systems have also enabled the development of hybrid platforms that can co-deliver cytotoxic agents alongside immunostimulatory cargo, a combinatorial capacity that fundamentally distinguishes vesicle-based strategies from conventional nanoparticle approaches and positions them as active modulators rather than passive carriers.

The clinical translation of these technologies requires careful consideration of key challenges, including large-scale production, standardization, and the maintenance of functional stability. Achieving consistent cargo loading, precise targeting, and efficient intracellular delivery remains essential for maximizing therapeutic efficacy. Addressing these challenges will require interdisciplinary efforts that combine advances in cell biology, materials science, and bioengineering. By incorporating patient-specific cells, high-resolution profiling, and data-driven design, vesicle-based therapies are evolving toward personalized, adaptive strategies. In this context, M1-like macrophage-derived vesicles and their engineered variants function as more than just delivery vehicles; they act as active, multi-dimensional modulators of the TME. Utilizing this potential will not only require continued innovation in vesicle engineering but also the establishment of translational frameworks that connect basic science with clinical application. Thus, the ability of M1-like macrophage-derived vesicles to simultaneously act as drug carriers, immune stimulants, and microenvironment remodelers positions them as a highly versatile, biologically coherent platform for precision cancer therapy.

## Figures and Tables

**Table 1:** List of studies with extracellular vesicles (EVs) and artificial nanovesicles (ANVs) in targeted cancer drug delivery.

Year	EV Type	Size	Cargoes	Target	Animal	Outcome	Ref
2018	M1 Nanovesicle (Exosome mimetic)	~190 nm	Pro-inflammatory cytokines, miRNAs	M2 TAMs	Mouse (Tumor-bearing)	Repolarized M2 to M1 macrophages, boosted aPD-L1 checkpoint inhibition	[101]
2019	M1 Exosome	~120 nm	Paclitaxel	4T1 Breast Cancer	Mouse (Breast Cancer)	Enhanced anti-tumor activity via macrophage-mediated inflammation	[8]
2021	M1 Exosome (IL4R-targeted)	50–200 nm	miR-511–3p, siRNA (NF-κB p50)	IL4R + TAMs	Mouse (Breast & Lung)	Reprogrammed TAMs into M1-like macrophages; inhibited tumor growth; enhanced anti-tumor immunity	[102]
2020	M1 Exosome	~100–120 nm	miR-16-5p	PD-L1 in Gastric Cancer	Mouse (Gastric Cancer)	Activated T cell response, suppressed PD-L1 expression	[103]
2022	M1 Exosome	~100 nm	Docetaxel	Breast Cancer	Mouse (Breast Cancer)	Enhanced drug delivery and tumor inhibition	[104]
2022	M1 Exosome	~100 nm	Zinc Phthalocyanine (ZnPc)	Colon Cancer	Mouse (MC38 model)	Improved photodynamic therapy efficacy	[105]
2022	Exosome-Mimetic Nanovesicles	~100–120 nm	-	Cancer cells	Mouse (Tumor model)	Efficient tumor homing and enhanced anti-tumor immune response	[106]
2022	M1 Membrane-Coated Liposomes	~150 nm	DOX, TPI-1	Osteosarcoma	Mouse (Osteosarcoma)	Synergistic tumor suppression and reduced systemic toxicity	[107]
2023	M1 Exosome	~100 nm	Cisplatin	Ovarian Cancer	Mouse (Ovarian Cancer)	Enhanced drug accumulation and improved tumor regression	[108]
2023	Hybrid EV (M1-EV + Thermoresponsive liposome)	~120 nm	-	Melanoma	Mouse (Melanoma)	Controlled drug release and effective tumor suppression	[8]
2023	Exosome-Mimetic M1 Nanovesicles	~100–120 nm	Docosahexaenoic acid (DHA)	Hepatocellular carcinoma	Mouse (HCC)	Suppressed tumor growth and induced apoptosis	[109]
2024	Hybrid EV (M1-EV + Liposome)	~150 nm	REV, SR780Fe, RS17 peptide	4T1 Breast Cancer	Mouse (Breast Cancer)	Enhanced tumor targeting and improved immunogenic cell death	[8]
2024	M1 Exosome	~100 nm	Ce6, endogenous NO	Colorectal Cancer	Mouse (Orthotopic CRC)	Dual photodynamic and gas therapy with strong anti-tumor effects	[110]
2024	M1-NV-Coated Lipid Nanoparticles (M1-C-LNPs)	~100–150 nm	Bcl2-siRNA + cytokines	CT26 Colon Cancer	Mouse (Colon Cancer)	Induced apoptosis, activated NK/T cells, strong tumor suppression	[111]
2025	M1 Macrophage-Derived Extracellular Particles (EPs)	~120 nm	Caspase-activating proteins, cytokines	MDA-MB-231 (TNBC)	Not specified (in vitro)	Induced apoptosis via caspase 3/7 activation in triple-negative breast cancer	[112]

EV: Extracellular Vesicle; NV: Nanovesicle; EP: Extracellular Particle; LNP: Lipid Nanoparticle; DOX: Doxorubicin; TPI-1: Triosephosphate Isomerase Inhibitor 1; ZnPc: Zinc Phthalocyanine; Ce6: Chlorin e6; DHA: Docosahexaenoic Acid; REV: Responsive Exosome Vesicle; siRNA: Small Interfering RNA; miRNA: MicroRNA; miR-511–3p: microRNA-511–3p; NF-κB: Nuclear Factor kappa-light-chain-enhancer of activated B cells; p50: NF-κB subunit p50; ROCK2: Rho-associated Coiled-Coil Containing Protein Kinase 2; IL4RPep-1: Interleukin-4 Receptor Binding Peptide 1; DOPE-PEG-NHS: 1,2-dioleoyl-sn-glycero-3-phosphoethanolamine-PEG-N-hydroxysuccinimide; CTRL: Control; IFN-γ: Interferon gamma; IL: Interleukin; TGF-β: Transforming Growth Factor beta; iNOS: inducible Nitric Oxide Synthase; M1: Classically Activated (Pro-inflammatory) Macrophage; M2: Alternatively Activated (Anti-inflammatory) Macrophage; TAMs: Tumor-Associated Macrophages; IL4R: Interleukin-4 Receptor; PD-L1: Programmed Death-Ligand 1; Bcl2: B-cell Lymphoma 2; 4T1: Mouse Breast Cancer Cell Line; LLC: Lewis Lung Carcinoma; CT26: Mouse Colon Carcinoma Cell Line; MC38: Murine Colon Adenocarcinoma Cell Line; MDA-MB-231: Human Triple-Negative Breast Cancer Cell Line; HEK293: Human Embryonic Kidney 293 Cell Line; TME: Tumor Microenvironment; MDSCs: Myeloid-Derived Suppressor Cells; Tregs: Regulatory T Cells; DCs: Dendritic Cells; CD: Cluster of Differentiation; CAR: Chimeric Antigen Receptor; CTL: Cytotoxic T Lymphocyte; aPD-L1: anti-PD-L1 antibody.

## Data Availability

No new data were created or analyzed in this study.

## References

[1] Anand U., Dey A., Chandel A.K.S., Sanyal R., Mishra A., Pandey D.K., De Falco V., Upadhyay A., Kandimalla R., Chaudhary A. (2023). Cancer Chemotherapy and Beyond: Current Status, Drug Candidates, Associated Risks and Progress in Targeted Therapeutics. Genes Dis..

[2] Runa F., Hamalian S., Meade K., Shisgal P., Gray P.C., Kelber J.A. (2017). Tumor Microenvironment Heterogeneity: Challenges and Opportunities. Curr. Mol. Biol. Rep..

[3] Neophytou C.M., Panagi M., Stylianopoulos T., Papageorgis P. (2021). The Role of Tumor Microenvironment in Cancer Metastasis: Molecular Mechanisms and Therapeutic Opportunities. Cancers.

[4] Christofides A., Strauss L., Yeo A., Cao C., Charest A., Boussiotis V.A. (2022). The Complex Role of Tumor-Infiltrating Macrophages. Nat. Immunol..

[5] Smith T.D., Tse M.J., Read E.L., Liu W.F. (2016). Regulation of Macrophage Polarization and Plasticity by Complex Activation Signals. Integr. Biol..

[6] Binnewies M., Roberts E.W., Kersten K., Chan V., Fearon D.F., Merad M., Coussens L.M., Gabrilovich D.I., Ostrand-Rosenberg S., Hedrick C.C. (2018). Understanding the Tumor Immune Microenvironment (TIME) for Effective Therapy. Nat. Med..

[7] Gangadaran P., Khan F., Rajendran R.L., Onkar A., Goenka A., Ahn B. (2024). Unveiling Invisible Extracellular Vesicles: Cutting-Edge Technologies for Their in Vivo Visualization. WIREs Nanomed. Nanobiotechnol..

[8] Koh H.B., Kim H.J., Kang S.-W., Yoo T.-H. (2023). Exosome-Based Drug Delivery: Translation from Bench to Clinic. Pharmaceutics.

[9] Krzyszczyk P., Schloss R., Palmer A., Berthiaume F. (2018). The Role of Macrophages in Acute and Chronic Wound Healing and Interventions to Promote Pro-wound Healing Phenotypes. Front. Physiol..

[10] Xu X., Gong X., Wang Y., Li J., Wang H., Wang J., Sha X., Li Y., Zhang Z. (2020). Reprogramming Tumor Associated Macrophages toward M1 Phenotypes with Nanomedicine for Anticancer Immunotherapy. Adv. Ther..

[11] Van Dalen F.J., Van Stevendaal M.H.M.E., Fennemann F.L., Verdoes M., Ilina O. (2018). Molecular Repolarisation of Tumour-Associated Macrophages. Molecules.

[12] Na Y.R., Yoon Y.N., Son D., Jung D., Gu G.J., Seok S.H. (2015). Consistent Inhibition of Cyclooxygenase Drives Macrophages towards the Inflammatory Phenotype. PLoS ONE.

[13] Pei Y., Yeo Y. (2016). Drug Delivery to Macrophages: Challenges and Opportunities. J. Control. Release.

[14] Yang Y., Hong Y., Cho E., Kim G.B., Kim I. (2018). Extracellular Vesicles as a Platform for Membrane-Associated Therapeutic Protein Delivery. J. Extracell. Vesicles.

[15] Wang L., Wang W., Hu D., Liang Y., Liu Z., Zhong T., Wang X. (2024). Tumor-Derived Extracellular Vesicles Regulate Macrophage Polarization: Role and Therapeutic Perspectives. Front. Immunol..

[16] Goenka A., Khan F., Verma B., Sinha P., Dmello C.C., Jogalekar M.P., Gangadaran P., Ahn B. (2023). Tumor Microenvironment Signaling and Therapeutics in Cancer Progression. Cancer Commun..

[17] Liu C., Feng Q., Sun J. (2019). Lipid Nanovesicles by Microfluidics: Manipulation, Synthesis, and Drug Delivery. Adv. Mater..

[18] Hirayama D., Iida T., Nakase H. (2017). The Phagocytic Function of Macrophage-Enforcing Innate Immunity and Tissue Homeostasis. Int. J. Mol. Sci..

[19] Locati M., Curtale G., Mantovani A. (2020). Diversity, Mechanisms, and Significance of Macrophage Plasticity. Annu. Rev. Pathol. Mech. Dis..

[20] Bercovici N., Guérin M.V., Trautmann A., Donnadieu E. (2019). The Remarkable Plasticity of Macrophages: A Chance to Fight Cancer. Front. Immunol..

[21] Epelman S., Lavine K.J., Randolph G.J. (2014). Origin and Functions of Tissue Macrophages. Immunity.

[22] Guan F., Wang R., Yi Z., Luo P., Liu W., Xie Y., Liu Z., Xia Z., Zhang H., Cheng Q. (2025). Tissue Macrophages: Origin, Heterogenity, Biological Functions, Diseases and Therapeutic Targets. Signal Transduct. Target. Ther..

[23] Orekhov A.N., Orekhova V.A., Nikiforov N.G., Myasoedova V.A., Grechko A.V., Romanenko E.B., Zhang D., Chistiakov D.A. (2019). Monocyte Differentiation and Macrophage Polarization. Vessel Plus.

[24] Kvedaraite E., Lourda M., Mouratidou N., Düking T., Padhi A., Moll K., Czarnewski P., Sinha I., Xagoraris I., Kokkinou E. (2024). Intestinal Stroma Guides Monocyte Differentiation to Macrophages Through GM-CSF. Nat. Commun..

[25] Liu J., Wang M., Zhao Y. (2025). The Regulatory Network of Transcription Factors in Macrophage Polarization. ImmunoTargets Ther..

[26] Stout R.D., Suttles J. (2004). Functional plasticity of macrophages: Reversible adaptation to changing microenvironments. J. Leukoc. Biol..

[27] Shapouri-Moghaddam A., Mohammadian S., Vazini H., Taghadosi M., Esmaeili S., Mardani F., Seifi B., Mohammadi A., Afshari J.T., Sahebkar A. (2018). Macrophage plasticity, polarization, and function in health and disease. J. Cell. Physiol..

[28] Nailwal N.P., Doshi G.M. (2021). Role of intracellular signaling pathways and their inhibitors in the treatment of inflammation. Inflammopharmacology.

[29] Malyshev I., Malyshev Y. (2015). Current Concept and Update of the Macrophage Plasticity Concept: Intracellular Mechanisms of Reprogramming and M3 Macrophage “Switch” Phenotype. BioMed Res. Int..

[30] Caldwell B.A., Li L. (2024). Epigenetic Regulation of Innate Immune Dynamics During Inflammation. J. Leukoc. Biol..

[31] Bao W., Qu X., Wang Y., Huang D., Zhang H., Dong M., Sun H., Yang Z., Li X. (2026). Tumor-Associated Macrophages as Therapeutic Targets: Deciphering Interaction Networks and Advancing Clinical Translation. MedComm.

[32] Stout R.D., Watkins S.K., Suttles J. (2009). Functional Plasticity of Macrophages: In Situ Reprogramming of Tumor-Associated Macrophages. J. Leukoc. Biol..

[33] Wang Q., Ma W. (2025). Revisiting TAM Polarization: Beyond M1- and M2-Type TAM Toward Clinical Precision in Macrophage-Targeted Therapy. Exp. Mol. Pathol..

[34] Merecz-Sadowska A., Sitarek P., Śliwiński T., Zajdel R. (2020). Anti-Inflammatory Activity of Extracts and Pure Compounds Derived from Plants via Modulation of Signaling Pathways, Especially PI3K/AKT in Macrophages. Int. J. Mol. Sci..

[35] Wynn T.A., Vannella K.M. (2016). Macrophages in Tissue Repair, Regeneration, and Fibrosis. Immunity.

[36] Sohrabi S., Alipour S., Ghahramanipour Z., Masoumi J., Baradaran B. (2024). STAT Signaling Pathways in Immune Cells and Their Associated Mechanisms in Cancer Pathogenesis. BioImpacts.

[37] Skytthe M.K., Graversen J.H., Moestrup S.K. (2020). Targeting of CD163+ Macrophages in Inflammatory and Malignant Diseases. Int. J. Mol. Sci..

[38] Rőszer T. (2015). Understanding the Mysterious M2 Macrophage through Activation Markers and Effector Mechanisms. Mediat. Inflamm..

[39] Strizova Z., Benesova I., Bartolini R., Novysedlak R., Cecrdlova E., Foley L.K., Striz I. (2023). M1/M2 Macrophages and Their Overlaps—Myth or Reality?. Clin. Sci..

[40] Viola A., Munari F., Sánchez-Rodríguez R., Scolaro T., Castegna A. (2019). The Metabolic Signature of Macrophage Responses. Front. Immunol..

[41] Zeng W., Li F., Jin S., Ho P.-C., Liu P.-S., Xie X. (2023). Functional Polarization of Tumor-Associated Macrophages Dictated by Metabolic Reprogramming. J. Exp. Clin. Cancer Res..

[42] Fu X., Pang M., Wang Z., Wang H. (2025). Macrophage Polarization in the Tumor Microenvironment of Hepatocellular Carcinoma: From Mechanistic Insights to Translational Therapies. Cancer Control.

[43] Xie C., Liu C., Wu B., Lin Y., Ma T., Xiong H., Wang Q., Li Z., Ma C., Tu Z. (2016). Effects of IRF1 and IFN-Β Interaction on the M1 Polarization of Macrophages and Its Antitumor Function. Int. J. Mol. Med..

[44] Zhang H., Liu L., Liu J., Dang P., Hu S., Yuan W., Sun Z., Liu Y., Wang C. (2023). Roles of Tumor-Associated Macrophages in Anti-PD-1/PD-L1 Immunotherapy for Solid Cancers. Mol. Cancer.

[45] Jurewicz M.M., Stern L.J. (2019). Class II MHC Antigen Processing in Immune Tolerance and Inflammation. Immunogenetics.

[46] Mantegazza A.R., Magalhaes J.G., Amigorena S., Marks M.S. (2013). Presentation of Phagocytosed Antigens by MHC Class I and II. Traffic.

[47] Lanier L.L., O’Fallon S., Somoza C., Phillips J.H., Linsley P.S., Okumura K., Ito D., Azuma M. (1995). CD80 (B7) and CD86 (B70) Provide Similar Costimulatory Signals for T Cell Proliferation, Cytokine Production, and Generation of CTL. J. Immunol..

[48] Gao Y., Li L., Zheng Y., Zhang W., Niu B., Li Y. (2022). Monoclonal Antibody Daratumumab Promotes Macrophage-Mediated Anti-Myeloma Phagocytic Activity Via Engaging FC Gamma Receptor and Activation of Macrophages. Mol. Cell. Biochem..

[49] Chen S., Lai S.W.T., Brown C.E., Feng M. (2021). Harnessing and Enhancing Macrophage Phagocytosis for Cancer Therapy. Front. Immunol..

[50] Chen S., Saeed A.F.U.H., Liu Q., Jiang Q., Xu H., Xiao G.G., Rao L., Duo Y. (2023). Macrophages in Immunoregulation and Therapeutics. Signal Transduct. Target. Ther..

[51] Ullrich K.A.-M., Schulze L.L., Paap E.-M., Müller T.M., Neurath M.F., Zundler S. (2020). Immunology of IL-12: An update on functional activities and implications for disease. EXCLI J..

[52] Laha D., Grant R., Mishra P., Nilubol N. (2021). The Role of Tumor Necrosis Factor in Manipulating the Immunological Response of Tumor Microenvironment. Front. Immunol..

[53] Wilburn W.J., Gabure S., Whalen M.M. (2024). Interleukin 1β and Interleukin 6 Production in Human Immune Cells Is Stimulated by the Antibacterial Compound Triclosan. Arch. Toxicol..

[54] Marcovecchio P.M., Thomas G., Salek-Ardakani S. (2021). CXCL9-Expressing Tumor-Associated Macrophages: New Players in the Fight Against Cancer. J. Immunother. Cancer.

[55] Fujii J., Osaki T. (2023). Involvement of Nitric Oxide in Protecting against Radical Species and Autoregulation of M1-Polarized Macrophages through Metabolic Remodeling. Molecules.

[56] Griess B., Mir S., Datta K., Teoh-Fitzgerald M. (2020). Scavenging Reactive Oxygen Species Selectively Inhibits M2 Macrophage Polarization and Their Pro-Tumorigenic Function in Part, Via Stat3 Suppression. Free Radic. Biol. Med..

[57] Tacconi S., Vari F., Sbarigia C., Vardanyan D., Longo S., Mura F., Angilè F., Jalabert A., Blangero F., Eljaafari A. (2024). M1-Derived Extracellular Vesicles Polarize Recipient Macrophages into M2-Like Macrophages and Alter Skeletal Muscle Homeostasis in a Hyper-Glucose Environment. Cell Commun. Signal..

[58] Guo R., Wang R., Zhang W., Li Y., Wang Y., Wang H., Li X., Song J. (2025). Macrophage Polarisation in the Tumour Microenvironment: Recent Research Advances and Therapeutic Potential of Different Macrophage Reprogramming. Cancer Control.

[59] Mijatović S., Savić-Radojević A., Plješa-Ercegovac M., Simić T., Nicoletti F., Maksimović-Ivanić D. (2020). The Double-Faced Role of Nitric Oxide and Reactive Oxygen Species in Solid Tumors. Antioxidants.

[60] Khan F.H., Dervan E., Bhattacharyya D.D., McAuliffe J.D., Miranda K.M., Glynn S.A. (2020). The Role of Nitric Oxide in Cancer: Master Regulator or NOt?. Int. J. Mol. Sci..

[61] Canton M., Sánchez-Rodríguez R., Spera I., Venegas F.C., Favia M., Viola A., Castegna A. (2021). Reactive Oxygen Species in Macrophages: Sources and Targets. Front. Immunol..

[62] Jakubzick C.V., Randolph G.J., Henson P.M. (2017). Monocyte Differentiation and Antigen-Presenting Functions. Nat. Rev. Immunol..

[63] Vackova J., Polakova I., Johari S.D., Smahel M. (2021). CD80 Expression on Tumor Cells Alters Tumor Microenvironment and Efficacy of Cancer Immunotherapy by CTLA-4 Blockade. Cancers.

[64] Qureshi O.S., Zheng Y., Nakamura K., Attridge K., Manzotti C., Schmidt E.M., Baker J., Jeffery L.E., Kaur S., Briggs Z. (2011). Trans-Endocytosis of CD80 and CD86: A Molecular Basis for the Cell-Extrinsic Function of CTLA-4. Science.

[65] Basu A., Ramamoorthi G., Albert G., Gallen C., Beyer A., Snyder C., Koski G., Disis M.L., Czerniecki B.J., Kodumudi K. (2021). Differentiation and Regulation of TH Cells: A Balancing Act for Cancer Immunotherapy. Front. Immunol..

[66] Yang Y., Li S., To K.K.W., Zhu S., Wang F., Fu L. (2025). Tumor-Associated Macrophages Remodel the Suppressive Tumor Immune Microenvironment and Targeted Therapy for Immunotherapy. J. Exp. Clin. Cancer Res..

[67] Huang R., Kang T., Chen S. (2024). The Role of Tumor-Associated Macrophages in Tumor Immune Evasion. J. Cancer Res. Clin. Oncol..

[68] Yang Y., Yang F., Huang Z., Li Y., Shi H., Sun Q., Ma Y., Wang Y., Zhang Y., Yang S. (2023). T Cells, NK Cells, and Tumor-Associated Macrophages in Cancer Immunotherapy and the Current State of the Art of Drug Delivery Systems. Front. Immunol..

[69] Helmin-Basa A., Gackowska L., Balcerowska S., Ornawka M., Naruszewicz N., Wiese-Szadkowska M. (2022). The Application of the Natural Killer Cells, Macrophages and Dendritic Cells in Treating Various Types of Cancer. Phys. Sci. Rev..

[70] Hussein A., Stamova S., Xydia M., Beckhove P. (2024). Hand in Hand to Successful Immunotherapy: CD8+ T Cells and M1-Like Macrophages Swap the Baton. Cancer Cell.

[71] Van Niel G., Carter D.R.F., Clayton A., Lambert D.W., Raposo G., Vader P. (2022). Challenges and Directions in Studying Cell–Cell Communication by Extracellular Vesicles. Nat. Rev. Mol. Cell Biol..

[72] Meng W., He C., Hao Y., Wang L., Li L., Zhu G. (2020). Prospects and Challenges of Extracellular Vesicle-Based Drug Delivery System: Considering Cell Source. Drug Deliv..

[73] Liu C., Wang Y., Li L., He D., Chi J., Li Q., Wu Y., Zhao Y., Zhang S., Wang L. (2022). Engineered Extracellular Vesicles and Their Mimetics for Cancer Immunotherapy. J. Control. Release.

[74] Huang J., Chen H., Li N., Liu P., Yang J., Zhao Y. (2025). Emerging Technologies Towards Extracellular Vesicles Large-Scale Production. Bioact. Mater..

[75] Livshits M.A., Khomyakova E., Evtushenko E.G., Lazarev V.N., Kulemin N.A., Semina S.E., Generozov E.V., Govorun V.M. (2015). Isolation of Exosomes by Differential Centrifugation: Theoretical Analysis of a Commonly Used Protocol. Sci. Rep..

[76] Duong P., Chung A., Bouchareychas L., Raffai R.L. (2019). Cushioned-Density Gradient Ultracentrifugation (C-DGUC) Improves the Isolation Efficiency of Extracellular Vesicles. PLoS ONE.

[77] Shami-shah A., Travis B.G., Walt D.R. (2023). Advances in Extracellular Vesicle Isolation Methods: A Path Towards Cell-Type Specific EV Isolation. Extracell. Vesicles Circ. Nucleic Acids.

[78] Monguió-Tortajada M., Gálvez-Montón C., Bayes-Genis A., Roura S., Borràs F.E. (2019). Extracellular Vesicle Isolation Methods: Rising Impact of Size-Exclusion Chromatography. Cell. Mol. Life Sci..

[79] Singh S.M., Furman R., Singh R.K., Balakrishnan G., Chennamsetty N., Tao L., Li Z. (2021). Size Exclusion Chromatography for the Characterization and Quality Control of Biologics. J. Liq. Chromatogr. Relat. Technol..

[80] Gaillard M., Thuaire A., Nonglaton G., Agache V., Roupioz Y., Raillon C. (2020). Biosensing Extracellular Vesicles: Contribution of Biomolecules in Affinity-Based Methods for Detection and Isolation. Analyst.

[81] Ströhle G., Gan J., Li H. (2022). Affinity-Based Isolation of Extracellular Vesicles and the Effects on Downstream Molecular Analysis. Anal. Bioanal. Chem..

[82] Clos-Sansalvador M., Monguió-Tortajada M., Roura S., Franquesa M., Borràs F.E. (2022). Commonly Used Methods for Extracellular Vesicles’ Enrichment: Implications in Downstream Analyses and Use. Eur. J. Cell Biol..

[83] Zhang S., Deng J., Li J., Tian F., Liu C., Fang L., Sun J. (2022). Advanced Microfluidic Technologies for Isolating Extracellular Vesicles. TrAC Trends Anal. Chem..

[84] Meggiolaro A., Moccia V., Brun P., Pierno M., Mistura G., Zappulli V., Ferraro D. (2022). Microfluidic Strategies for Extracellular Vesicle Isolation: Towards Clinical Applications. Biosensors.

[85] Zhang Y., Lan M., Chen Y. (2024). Minimal Information for Studies of Extracellular Vesicles (MISEV): Ten-Year Evolution (2014–2023). Pharmaceutics.

[86] Welsh J.A., Goberdhan D.C.I., O’Driscoll L., Buzas E.I., Blenkiron C., Bussolati B., Cai H., Di Vizio D., Driedonks T.A.P., Erdbrügger U. (2024). Minimal Information for Studies of Extracellular Vesicles (MISEV2023): From Basic to Advanced Approaches. J. Extracell. Vesicles.

[87] Eshghjoo S., Kim D.M., Jayaraman A., Sun Y., Alaniz R.C. (2021). A Comprehensive High-Efficiency Protocol for Isolation, Culture, Polarization, and Glycolytic Characterization of Bone Marrow-Derived Macrophages. J. Vis. Exp..

[88] Shi Y., Luo P., Wang W., Horst K., Bläsius F., Relja B., Xu D., Hildebrand F., Greven J. (2020). M1 But Not M0 Extracellular Vesicles Induce Polarization of RAW264.7 Macrophages Via the TLR4-NFκB Pathway In Vitro. Inflammation.

[89] Mäki-Mantila K., Niskanen E.A., Kainulainen K., Pardas L.P., Aaltonen N., Wahbi W., Takabe P., Rönkä A., Rilla K., Pasonen-Seppänen S. (2025). Extracellular Vesicles Derived from Pro-Inflammatory M1 Macrophages Induce an Inflammatory and Invasive Phenotype in Melanoma Cells. Cell Commun. Signal..

[90] Kronstadt S.M., Van Heyningen L.H., Aranda A., Jay S.M. (2023). Assessment of Anti-Inflammatory Bioactivity of Extracellular Vesicles Is Susceptible to Error Via Media Component Contamination. Cytotherapy.

[91] Arteaga-Blanco L.A., Mojoli A., Monteiro R.Q., Sandim V., Menna-Barreto R.F.S., Pereira-Dutra F.S., Bozza P.T., Resende R.D.O., Bou-Habib D.C. (2020). Characterization and Internalization of Small Extracellular Vesicles Released by Human Primary Macrophages Derived from Circulating Monocytes. PLoS ONE.

[92] Patras L., Ionescu A.E., Munteanu C., Hajdu R., Kosa A., Porfire A., Licarete E., Rauca V.F., Sesarman A., Luput L. (2022). Trojan Horse Treatment Based on PEG-Coated Extracellular Vesicles to Deliver Doxorubicin to Melanoma In-Vitro and In-Vivo. Cancer Biol. Ther..

[93] Xu X., Xu L., Wang J., Wen C., Xia J., Zhang Y., Liang Y. (2024). Bioinspired Cellular Membrane-Derived Vesicles for mRNA Delivery. Theranostics.

[94] Cho N.-J., Hwang L., Solandt J., Frank C. (2013). Comparison of Extruded and Sonicated Vesicles for Planar Bilayer Self-Assembly. Materials.

[95] Li C., Luo D., Peng C., Luo Q., Xu W., Zhou J., Su W., Wu W., Wang Y.-F. (2026). Nitrogen Cavitation Enables Rapid and High-Yield Preparation of Functional Cell-Membrane-Derived Vesicles. Nanoscale Adv..

[96] Gao J., Dong X., Wang Z. (2020). Generation, Purification and Engineering of Extracellular Vesicles and Their Biomedical Applications. Methods.

[97] Li Z., Li M., Lu J. (2026). Biomimetic Liposomes in Drug Delivery: From Design Mechanisms to Applications. Chem. Soc. Rev..

[98] Chugh V., Vijaya Krishna K., Pandit A. (2021). Cell Membrane-Coated Mimics: A Methodological Approach for Fabrication, Characterization for Therapeutic Applications, and Challenges for Clinical Translation. ACS Nano.

[99] Du S., Guan Y., Xie A., Yan Z., Gao S., Li W., Rao L., Chen X., Chen T. (2023). Extracellular Vesicles: A Rising Star for Therapeutics and Drug Delivery. J. Nanobiotechnol..

[100] Chen Y., Zhao Y., Yin Y., Jia X., Mao L. (2021). Mechanism of Cargo Sorting into Small Extracellular Vesicles. Bioengineered.

[101] Choo Y.W., Kang M., Kim H.Y., Han J., Kang S., Lee J.-R., Jeong G.-J., Kwon S.P., Song S.Y., Go S. (2018). M1 Macrophage-Derived Nanovesicles Potentiate the Anticancer Efficacy of Immune Checkpoint Inhibitors. ACS Nano.

[102] Gunassekaran G.R., Poongkavithai Vadevoo S.M., Baek M.-C., Lee B. (2021). M1 Macrophage Exosomes Engineered to Foster M1 Polarization and Target the IL-4 Receptor Inhibit Tumor Growth by Reprogramming Tumor-Associated Macrophages into M1-Like Macrophages. Biomaterials.

[103] Li Z., Suo B., Long G., Gao Y., Song J., Zhang M., Feng B., Shang C., Wang D. (2020). Exosomal miRNA-16-5p Derived From M1 Macrophages Enhances T Cell-Dependent Immune Response by Regulating PD-L1 in Gastric Cancer. Front. Cell Dev. Biol..

[104] Zhao Y., Zheng Y., Zhu Y., Li H., Zhu H., Liu T. (2022). Docetaxel-Loaded M1 Macrophage-Derived Exosomes for a Safe and Efficient Chemoimmunotherapy of Breast Cancer. J. Nanobiotechnol..

[105] Huis In ‘T Veld R.V., Lara P., Jager M.J., Koning R.I., Ossendorp F., Cruz L.J. (2022). M1-Derived Extracellular Vesicles Enhance Photodynamic Therapy and Promote Immunological Memory in Preclinical Models of Colon Cancer. J. Nanobiotechnol..

[106] Baek S., Jeon M., Jung H.N., Lee W., Hwang J.-E., Lee J.S., Choi Y., Im H.-J. (2022). M1 Macrophage-Derived Exosome-Mimetic Nanovesicles with an Enhanced Cancer Targeting Ability. ACS Appl. Bio Mater..

[107] Wu F., Xu J., Chen Z., Jin M., Li X., Li J., Wang Z., Li J., Lu Q. (2022). Macrophage Membrane-Coated Liposomes as Controlled Drug Release Nanocarriers for Precision Treatment of Osteosarcoma. ACS Appl. Nano Mater..

[108] Zhang X., Wang J., Liu N., Wu W., Li H., Lu W., Guo X. (2023). Umbilical Cord Blood-Derived M1 Macrophage Exosomes Loaded with Cisplatin Target Ovarian Cancer In Vivo and Reverse Cisplatin Resistance. Mol. Pharm..

[109] Meng M., Zhang X., Li Q., Han J., Chen Y., Qiao H., Yang Y., Huang X. (2023). Engineering M1-Derived Nanovesicles Loading with Docosahexaenoic Acid Synergizes Ferroptosis and Immune Activation for Treating Hepatocellular Carcinoma. Cancer Nanotechnol..

[110] Zhang R.-Y., Cheng K., Huang Z.-Y., Zhang X.-S., Li Y., Sun X., Yang X.-Q., Hu Y.-G., Hou X.-L., Liu B. (2024). M1 Macrophage-Derived Exosome for Reprograming M2 Macrophages and Combining Endogenous NO Gas Therapy with Enhanced Photodynamic Synergistic Therapy in Colorectal Cancer. J. Colloid Interface Sci..

[111] Shin H.E., Han J.-H., Shin S., Bae G.-H., Son B., Kim T.-H., Park H.H., Park C.G., Park W. (2024). M1-Polarized Macrophage-Derived Cellular Nanovesicle-Coated Lipid Nanoparticles for Enhanced Cancer Treatment Through Hybridization of Gene Therapy and Cancer Immunotherapy. Acta Pharm. Sin. B.

[112] Desai P., Kumari A., Al Abdullah S., Anwar A., Nowlin K., Dellinger K. (2025). M1 Macrophage-Derived Extracellular Particles Induce Cell Death in MDA-MB-231 Cells. Cancer Rep..

[113] Kim G., Jeon H., Chao A., Johnston J., Zhu R., Khong C., Liu Y., Liang M., Lu X., Wang Y. (2026). M1 Macrophage-Derived Small Extracellular Vesicles as Synergistic Nanotherapeutics: Harnessing Intrinsic Anticancer Activity and Drug Delivery Capacity. J. Extracell. Vesicles.

[114] Walker S., Busatto S., Pham A., Tian M., Suh A., Carson K., Quintero A., Lafrence M., Malik H., Santana M.X. (2019). Extracellular Vesicle-Based Drug Delivery Systems for Cancer Treatment. Theranostics.

[115] Brezgin S., Danilik O., Yudaeva A., Kachanov A., Kostyusheva A., Karandashov I., Ponomareva N., Zamyatnin A.A., Parodi A., Chulanov V. (2024). Basic Guide for Approaching Drug Delivery with Extracellular Vesicles. Int. J. Mol. Sci..

[116] Liang Y., Duan L., Lu J., Xia J. (2021). Engineering Exosomes for Targeted Drug Delivery. Theranostics.

[117] Zhao F., Zhao Y., Liu Y., Chang X., Chen C., Zhao Y. (2011). Cellular Uptake, Intracellular Trafficking, and Cytotoxicity of Nanomaterials. Small.

[118] Gandek T.B., Van Der Koog L., Nagelkerke A. (2023). A Comparison of Cellular Uptake Mechanisms, Delivery Efficacy, and Intracellular Fate between Liposomes and Extracellular Vesicles. Adv. Healthc. Mater..

[119] Lamichhane T.N., Jeyaram A., Patel D.B., Parajuli B., Livingston N.K., Arumugasaamy N., Schardt J.S., Jay S.M. (2016). Oncogene Knockdown via Active Loading of Small RNAs into Extracellular Vesicles by Sonication. Cell. Mol. Bioeng..

[120] Sun L., Fan M., Huang D., Li B., Xu R., Gao F., Chen Y. (2021). Clodronate-Loaded Liposomal and Fibroblast-Derived Exosomal Hybrid System for Enhanced Drug Delivery to Pulmonary Fibrosis. Biomaterials.

[121] Rankin-Turner S., Vader P., O’Driscoll L., Giebel B., Heaney L.M., Davies O.G. (2021). A Call for the Standardised Reporting of Factors Affecting the Exogenous Loading of Extracellular Vesicles with Therapeutic Cargos. Adv. Drug Deliv. Rev..

[122] Wen Z., Liu C., Teng Z., Jin Q., Liao Z., Zhu X., Huo S. (2023). Ultrasound Meets the Cell Membrane: For Enhanced Endocytosis and Drug Delivery. Nanoscale.

[123] Buntsma N.C., Roos Y.B.W.E.M., Van Leeuwen T.G., Van Der Pol E., Nieuwland R. (2022). EDTA Stabilizes the Concentration of Platelet-Derived Extracellular Vesicles During Blood Collection and Handling. Platelets.

[124] Isazadeh H., Oruji F., Shabani S., Behroozi J., Nasiri H., Isazadeh A., Akbari M. (2023). Advances in siRNA Delivery Approaches in Cancer Therapy: Challenges and Opportunities. Mol. Biol. Rep..

[125] Wang J., Yin B., Lian J., Wang X. (2024). Extracellular Vesicles as Drug Delivery System for Cancer Therapy. Pharmaceutics.

[126] Li Y.-J., Wu J.-Y., Liu J., Xu W., Qiu X., Huang S., Hu X.-B., Xiang D.-X. (2021). Artificial Exosomes for Translational Nanomedicine. J. Nanobiotechnol..

[127] Pisani S., Di Martino D., Cerri S., Genta I., Dorati R., Bertino G., Benazzo M., Conti B. (2023). Investigation and Comparison of Active and Passive Encapsulation Methods for Loading Proteins into Liposomes. Int. J. Mol. Sci..

[128] Zhao P., Tian Y., Lu Y., Zhang J., Tao A., Xiang G., Liu Y. (2022). Biomimetic Calcium Carbonate Nanoparticles Delivered IL-12 mRNA for Targeted Glioblastoma Sono-Immunotherapy by Ultrasound-Induced Necroptosis. J. Nanobiotechnol..

[129] Tang Z., Tang C., Sun C., Ying X., Shen R. (2022). M1 Macrophage-Derived Exosomes Synergistically Enhance the Anti-Bladder Cancer Effect of Gemcitabine. Aging.

[130] Li J., Li N., Wang J. (2023). M1 Macrophage-Derived Exosome-Encapsulated Cisplatin Can Enhance Its Anti-Lung Cancer Effect. Minerva Med..

[131] Balaraman A.K., Arockia Babu M., Afzal M., Sanghvi G., Rekha M.M., Gupta S., Rana M., Ali H., Goyal K., Subramaniyan V. (2025). Exosome-Based miRNA Delivery: Transforming Cancer Treatment with Mesenchymal Stem Cells. Regen. Ther..

[132] Zeng H., Guo S., Ren X., Wu Z., Liu S., Yao X. (2023). Current Strategies for Exosome Cargo Loading and Targeting Delivery. Cells.

[133] Torabi C., Choi S.-E., Pisanic T.R., Paulaitis M., Hur S.C. (2024). Streamlined miRNA Loading of Surface Protein-Specific Extracellular Vesicle Subpopulations Through Electroporation. Biomed. Eng. Online.

[134] Setia A., Sahu R.K., Ray S., Widyowati R., Ekasari W., Saraf S. (2022). Advances in Hybrid Vesicular-based Drug Delivery Systems: Improved Biocompatibility, Targeting, Therapeutic Efficacy and Pharmacokinetics of Anticancer Drugs. Curr. Drug Metab..

[135] Ullmann K., Leneweit G., Nirschl H. (2021). How to Achieve High Encapsulation Efficiencies for Macromolecular and Sensitive APIs in Liposomes. Pharmaceutics.

[136] Trucillo P., Campardelli R., Reverchon E. (2017). Supercritical CO_2_ Assisted Liposomes Formation: Optimization of the Lipidic Layer for an Efficient Hydrophilic Drug Loading. J. CO2 Util..

[137] Gopaldass N., Roy Chowdhury S., Alves A.C., Michaillat Mayer L., Comte-Misérez V., Mayer A. (2026). Hybrid Endosomal Coats Contain Different Classes of Sorting Nexins. EMBO J..

[138] Buzas E.I. (2023). The Roles of Extracellular Vesicles in the Immune System. Nat. Rev. Immunol..

[139] Mohammadi F., Dikpati A., Bertrand N., Rudkowska I. (2024). Encapsulation of Conjugated Linoleic Acid and Ruminant *Trans* Fatty Acids to Study the Prevention of Metabolic Syndrome—A Review. Nutr. Rev..

[140] Rawat S., Arora S., Dhondale M.R., Khadilkar M., Kumar S., Agrawal A.K. (2025). Stability Dynamics of Plant-Based Extracellular Vesicles Drug Delivery. J. Xenobiot..

[141] Bala A., Elelu S.-A., Ibrahim G.O., Temitope I.A., Livinus M.U., Yasir A.M., Innocent M.O., Abdulsalam M. (2025). Peptide-Based Approaches for Biomolecule Encapsulation, Storage, and Preservation: A Comprehensive Review. Biol. Sci..

[142] Gurunathan S., Kang M.-H., Qasim M., Khan K., Kim J.-H. (2021). Biogenesis, Membrane Trafficking, Functions, and Next Generation Nanotherapeutics Medicine of Extracellular Vesicles. Int. J. Nanomed..

[143] Van Der Koog L., Gandek T.B., Nagelkerke A. (2022). Liposomes and Extracellular Vesicles as Drug Delivery Systems: A Comparison of Composition, Pharmacokinetics, and Functionalization. Adv. Healthc. Mater..

[144] Ghadami S., Dellinger K. (2023). The Lipid Composition of Extracellular Vesicles: Applications in Diagnostics and Therapeutic Delivery. Front. Mol. Biosci..

[145] Le Q.-V., Lee J., Lee H., Shim G., Oh Y.-K. (2021). Cell Membrane-Derived Vesicles for Delivery of Therapeutic Agents. Acta Pharm. Sin. B.

[146] Roerig J., Schulz-Siegmund M. (2023). Standardization Approaches for Extracellular Vesicle Loading with Oligonucleotides and Biologics. Small.

[147] Clares-Pedrero I., Rocha-Mulero A., Palma-Cobo M., Cardeñes B., Yáñez-Mó M., Cabañas C. (2024). Molecular Determinants Involved in the Docking and Uptake of Tumor-Derived Extracellular Vesicles: Implications in Cancer. Int. J. Mol. Sci..

[148] Tian J.-W., Fang Y.-H., Zhang H.-J., Yu Z.-L. (2025). The Dual Effects of Macrophage-Derived Extracellular Vesicles on Tumor Cell Behavior: Mechanisms and Clinical Potential. Front. Oncol..

[149] Kooijmans S.A.A., Fliervoet L.A.L., Van Der Meel R., Fens M.H.A.M., Heijnen H.F.G., Van Bergen En Henegouwen P.M.P., Vader P., Schiffelers R.M. (2016). PEGylated and Targeted Extracellular Vesicles Display Enhanced Cell Specificity and Circulation Time. J. Control. Release.

[150] Wiklander O.P.B., Mamand D.R., Mohammad D.K., Zheng W., Jawad Wiklander R., Sych T., Zickler A.M., Liang X., Sharma H., Lavado A. (2024). Antibody-Displaying Extracellular Vesicles for Targeted Cancer Therapy. Nat. Biomed. Eng..

[151] Xiao R., Zhou G., Wen Y., Ye J., Li X., Wang X. (2023). Recent Advances on Stimuli-Responsive Biopolymer-Based Nanocomposites for Drug Delivery. Compos. Part B Eng..

[152] Mulcahy L.A., Pink R.C., Carter D.R.F. (2014). Routes and Mechanisms of Extracellular Vesicle Uptake. J. Extracell. Vesicles.

[153] Kiss A.L., Botos E. (2009). Endocytosis *via* caveolae: Alternative pathway with distinct cellular compartments to avoid lysosomal degradation?. J. Cell. Mol. Med..

[154] Gangadaran P., Onkar A., Rajendran R.L., Goenka A., Oh J.M., Khan F., Nagarajan A.K., Muthu S., Krishnan A., Hong C.M. (2025). Noninvasive in Vivo Imaging of Macrophages: Understanding Tumor Microenvironments and Delivery of Therapeutics. Biomark. Res..

[155] Ribovski L., Joshi B., Gao J., Zuhorn I. (2023). Erratum: Breaking Free: Endocytosis and Endosomal Escape of Extracellular Vesicles. Extracell. Vesicles Circ. Nucleic Acids.

[156] Chen T., Chen D., Su W., Liang J., Liu X., Cai M. (2025). Extracellular Vesicles as Vital Players in Drug Delivery: A Focus on Clinical Disease Treatment. Front. Bioeng. Biotechnol..

[157] Chahal G.S., Helbig K.J., Parton R.G., Monson E.A. (2026). The Biology of Endosomal Escape: Strategies for Enhanced Delivery of Therapeutics. ACS Nano.

[158] Onkar A., Khan F., Goenka A., Rajendran R.L., Dmello C., Hong C.M., Mubin N., Gangadaran P., Ahn B.-C. (2024). Smart Nanoscale Extracellular Vesicles in the Brain: Unveiling their Biology, Diagnostic Potential, and Therapeutic Applications. ACS Appl. Mater. Interfaces.

[159] Pei D., Buyanova M. (2019). Overcoming Endosomal Entrapment in Drug Delivery. Bioconjug. Chem..

[160] Yin F., He Y., Qiao Y., Yan Y. (2025). Tumor-Derived Vesicles in Immune Modulation: Focus on Signaling Pathways. Front. Immunol..

[161] Liu H., Ouyang Z., Li S. (2024). Advances of M1 Macrophages-Derived Extracellular Vesicles in Tumor Therapy. Biomed. Pharmacother..

[162] Liu X., Kwon H., Li Z., Fu Y.-X. (2017). Is CD47 an Innate Immune Checkpoint for Tumor Evasion?. J. Hematol. Oncol. J. Hematol. Oncol..

[163] Wang X., Ding H., Li Z., Peng Y., Tan H., Wang C., Huang G., Li W., Ma G., Wei W. (2022). Exploration and Functionalization of M1-Macrophage Extracellular Vesicles for Effective Accumulation in Glioblastoma and Strong Synergistic Therapeutic Effects. Signal Transduct. Target. Ther..

[164] Kuang L., Wu L., Li Y. (2025). Extracellular Vesicles in Tumor Immunity: Mechanisms and Novel Insights. Mol. Cancer.

[165] Li S., Zhang J., Feng G., Jiang L., Chen Z., Xin W., Zhang X. (2022). The Emerging Role of Extracellular Vesicles from Mesenchymal Stem Cells and Macrophages in Pulmonary Fibrosis: Insights into miRNA Delivery. Pharmaceuticals.

[166] Kumar D.N., Chaudhuri A., Aqil F., Dehari D., Munagala R., Singh S., Gupta R.C., Agrawal A.K. (2022). Exosomes as Emerging Drug Delivery and Diagnostic Modality for Breast Cancer: Recent Advances in Isolation and Application. Cancers.

[167] Zhou W., Yang F., Zhang X. (2024). Roles of M1 Macrophages and Their Extracellular Vesicles in Cancer Therapy. Cells.

[168] Kim Y.K., Hong Y., Bae Y.R., Goo J., Kim S.A., Choi Y., Nam G.-H., Kwon M., Yun S.G., Lee G. (2022). Advantage of Extracellular Vesicles in Hindering the CD47 Signal for Cancer Immunotherapy. J. Control. Release.

[169] Silva R.M., Azevedo A.M., Bonifácio V.D.B., Fernandez-Becerra C., Pinto S.N. (2025). Unlocking the Potential of Extracellular Vesicles: One Stimulus Away from Clinical Implementation. Biomater. Sci..

[170] Han L., Song Y., Tong L., Sun J., Zhang X., Chen S., Li Y., Wang Z., Gao L., Zhu Q. (2025). Extracellular Vesicle Protein Panel Enables Early Lung Cancer Detection in a Large Clinical Cohort. J. Extracell. Vesicles.

[171] Kooijmans S.A.A., De Jong O.G., Schiffelers R.M. (2021). Exploring Interactions Between Extracellular Vesicles and Cells for Innovative Drug Delivery System Design. Adv. Drug Deliv. Rev..

[172] Ma Y., Brocchini S., Williams G.R. (2023). Extracellular Vesicle-Embedded Materials. J. Control. Release.

[173] Suk J.S., Xu Q., Kim N., Hanes J., Ensign L.M. (2016). PEGylation as a Strategy for Improving Nanoparticle-Based Drug and Gene Delivery. Adv. Drug Deliv. Rev..

[174] Hao Y., Ji Z., Zhou H., Wu D., Gu Z., Wang D., Ten Dijke P. (2023). Lipid-Based Nanoparticles as Drug Delivery Systems for Cancer Immunotherapy. MedComm.

[175] Yao C., Yu J., Xie D., Zhang S., Tao J., Kong L., Fang L., Zhu Q., Fang M. (2026). Source-Specific Extracellular Vesicle Functions and Engineering Strategies for Chronic Pain Management: A Comprehensive Review. Int. J. Nanomed..

[176] Gandham S., Su X., Wood J., Nocera A.L., Alli S.C., Milane L., Zimmerman A., Amiji M., Ivanov A.R. (2020). Technologies and Standardization in Research on Extracellular Vesicles. Trends Biotechnol..

[177] Taciak B., Grochowska A., Górczak M., Górka E., Skorzynski M., Białasek M., Rygiel T.P., Król M. (2025). Unveiling the Phenotypic Variability of Macrophages: Insights from Donor Diversity and Pooling Strategies. Int. J. Mol. Sci..

[178] Witwer K.W., Buzás E.I., Bemis L.T., Bora A., Lässer C., Lötvall J., Nolte-‘t Hoen E.N., Piper M.G., Sivaraman S., Skog J. (2013). Standardization of Sample Collection, Isolation and Analysis Methods in Extracellular Vesicle Research. J. Extracell. Vesicles.

[179] Buschmann D., Mussack V., Byrd J.B. (2021). Separation, Characterization, and Standardization of Extracellular Vesicles for Drug Delivery Applications. Adv. Drug Deliv. Rev..

[180] Li X., Li X., Tong L., Hu L., Hong Y., Zhou R., Li Z., Dong M., Hou J., Xu T. (2025). Systematic Evaluation of Isolation Techniques and Freeze-Thaw Effects on Plasma Extracellular Vesicle Heterogeneity and Subpopulation Profiling. J. Extracell. Biol..

[181] Wang Z., Zhou X., Kong Q., He H., Sun J., Qiu W., Zhang L., Yang M. (2024). Extracellular Vesicle Preparation and Analysis: A State-of-the-Art Review. Adv. Sci..

[182] Chen H., Li Q. (2025). Recent Advances in Scalable Exosome Production: Challenges and Innovations. Chin. J. Plast. Reconstr. Surg..

[183] Ma Y., Dong S., Wu A., Jeong S.D., Lee A.S., Jiang W., Kim B.Y.S. (2026). Engineering Challenges and Translational Opportunities in Emerging Gene Delivery Platforms. Nat. Biomed. Eng..

[184] Mukherjee A., Bisht B., Dutta S., Paul M.K. (2022). Current Advances in the Use of Exosomes, Liposomes, and Bioengineered Hybrid Nanovesicles in Cancer Detection and Therapy. Acta Pharmacol. Sin..

[185] Obata Y., Tajima S., Takeoka S. (2010). Evaluation of pH-Responsive Liposomes Containing Amino Acid-Based Zwitterionic Lipids for Improving Intracellular Drug Delivery in Vitro and in Vivo. J. Control. Release.

[186] Yang Y., Liu J., Liu J., Wei S., Kong X., Mu W., Liu Y., Zhang N. (2026). Advances in Immune Cell-Based Therapeutic Agents for the Treatment of Inflammation-Related Diseases. Acta Pharm. Sin. B.

[187] Hsu C.-Y., Askar S., Alshkarchy S.S., Nayak P.P., Attabi K.A.L., Khan M.A., Mayan J.A., Sharma M.K., Islomov S., Soleimani Samarkhazan H. (2025). AI-Driven Multi-Omics Integration in Precision Oncology: Bridging the Data Deluge to Clinical Decisions. Clin. Exp. Med..

[188] Shojaei-Ghahrizjani F., Tawil N., Meehan B., Montermini L., Khajeh M., Villa A.M., Rak J., Ciana P. (2026). Cancer-derived Extracellular Vesicles for Targeted Delivery of EGFRvIII siRNA to Glioblastoma, Comparison of siRNA Loading Methods and Efficiency. bioRxiv.

